# Pioneering noninvasive colorectal cancer detection with an AI-enhanced breath volatilomics platform

**DOI:** 10.7150/thno.94950

**Published:** 2024-07-08

**Authors:** Yongqian Liu, Yongyan Ji, Jian Chen, Yixuan Zhang, Xiaowen Li, Xiang Li

**Affiliations:** 1 Department of Environmental Science & Engineering, Fudan University, Shanghai 200438, P.R. China.; 2 Department of gastroenterology, Huadong hospital, Fudan University, Shanghai 200040, P.R. China.

**Keywords:** Colorectal cancer, Breath biomarker, Artificial Intelligence, Noninvasive detection, Gut microbiome.

## Abstract

**Background:** The sensitivity and specificity of current breath biomarkers are often inadequate for effective cancer screening, particularly in colorectal cancer (CRC). While a few exhaled biomarkers in CRC exhibit high specificity, they lack the requisite sensitivity for early-stage detection, thereby limiting improvements in patient survival rates.

**Methods:** In this study, we developed an advanced Mass Spectrometry-based volatilomics platform, complemented by an enhanced breath sampler. The platform integrates artificial intelligence (AI)-assisted algorithms to detect multiple volatile organic compounds (VOCs) biomarkers in human breath. Subsequently, we applied this platform to analyze 364 clinical CRC and normal exhaled samples.

**Results:** The diagnostic signatures, including 2-methyl, octane, and butyric acid, generated by the platform effectively discriminated CRC patients from normal controls with high sensitivity (89.7%), specificity (86.8%), and accuracy (AUC = 0.91). Furthermore, the metastatic signature correctly identified over 50% of metastatic patients who tested negative for carcinoembryonic antigen (CEA). Fecal validation indicated that elevated breath biomarkers correlated with an inflammatory response guided by Bacteroides fragilis in CRC.

**Conclusion:** This study introduces a sophisticated AI-aided Mass Spectrometry-based platform capable of identifying novel and feasible breath biomarkers for early-stage CRC detection. The promising results position the platform as an efficient noninvasive screening test for clinical applications, offering potential advancements in early detection and improved survival rates for CRC patients.

## Introduction

Colorectal cancer (CRC) is the third most prevalent cancer and the second most frequent cancer-related cause of death worldwide 1. Projections indicate that by 2030, global CRC cases will witness a 60% surge, reaching over 2 million new cases, and leading to around 1 million fatalities 2. Earlier detection of CRC could increase survival by an estimated 30 to 40%. Moreover, patient prognosis in CRC is predominantly influenced by the clinical stage at diagnosis, especially the presence of distant metastasis. Despite state-of-the-art computed tomography, fecal occult blood test and serum carcinoembryonic antigen (CEA) for patients with CRC, the rate of correct diagnosis is 40-65% and the rate of identifying metastases is 40-60% 3. Meanwhile, these traditional methods are invasive, time-consuming, expensive and may lead to complications. Given these challenges, it is urgent to introduce a novel diagnostic tool for precise identification of patients with preclinical and truly localized disease in CRC with higher patient compliance and low cost.

The breath serves as a valuable source for recognizing highly sensitive biomarkers as it promptly reflects bodily changes 3, 4. Moreover, volatile organic compounds (VOCs) composition in the breath is significantly simpler than that of serum or plasma, making the breath an optimal choice for analyses 5. Both our research and prior investigations have identified >180 VOCs in the human breath 6. Approximately 40% of these VOCs in the breath originate from those in plasma, and over 100 VOCs produced in the colorectum can be detected in the breath of healthy subjects 7. Thus, the breath is a feasible tool for identifying noninvasive biomarkers for CRC. However, large-scaled and effective application of breath biomarkers in clinical practice is often hampered by three challenges: (i) Lack of standardization in sample collection. Tedlar® and other polymer storage bags may lead to limited sensitivity due to collection of single exhaled breath and contamination with VOCs from bag 8, 9. (ii) Deficiency of a universal breath VOCs analysis method. The common analytical instruments were limited by inter-instrument variability, temporal stability and poor chemical selectivity, such as SIFT-MS and Electronic Nose 10, 11. (iii) Insufficiency of machine learning analytical methods to recognize reliable marker panel. Some models are of poor design and inadequate sample size, risking bias and overfitting 12. Hence, a comprehensive study including standardized methodology for breath analysis and biomarkers screening, has still been unavailable until now.

Here, we developed a prospective MS-based volatolomics platform combined with improved breath sampler for detection of CRC to directly address the challenges of standardization in breath sampling and analysis in translational clinical analyses. Then we utilized optimized artificial intelligence (AI)-based machine learning (ML) algorithms with vigorous feature selection to build diagnostic and metastatic models of 14 markers and 7 markers, respectively. The sensitivity and specificity of the models were evaluated in the clinically relevant cohort of healthy individuals and those with CRC in two stages, and compared with that of CEA. At last, the alteration of crucial breath VOCs is correlated in gut microbiome. This step-by-step research generates greatly precise non-invasive breath VOCs markers and demonstrates the promising role of breathomics in future CRC detection.

## Results

### Study design and clinical characteristics

**Figure 1:**
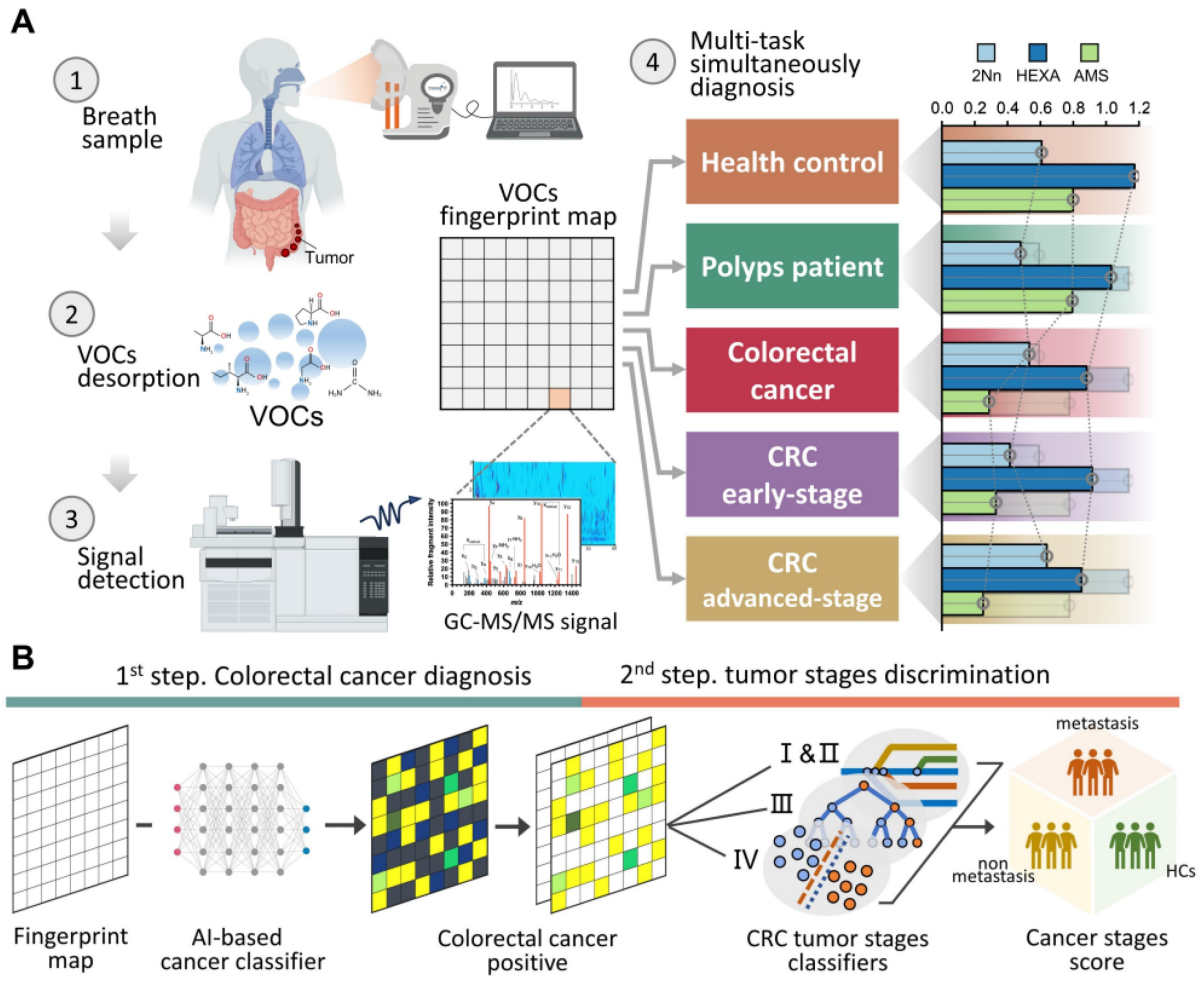
** One test-multi-CRC using VOCs-MS-AI. A.** Overview. Human breath with endogenous VOCs is collected using improved sampler. Signals were observed by TD-GC-MS/MS and analyzed by AI algorithms. The system outputs predictions about cancer presence and cancer metastasis. A histogram shows actual examples of the representative predicted results for each cancer status. **B.** AI framework. In the first step, diagnostic model is constructed through the multiple AI-based classifier results. In the second step, signatures extracted by the previous CRC classifier are analyzed, then a metastatic marker panel is generated using three types of feature selection algorithms. Cartoons were created with BioRender.com.

Figure 1A demonstrate a breath biopsy technique that merges AI and TD-GC × GC-QQQ MS to simultaneously diagnose CRC by untargeted analysis of exhaled VOCs. Our technique acquires GC-MS signals of endogenous VOCs, then analyzes them using robust machine learning algorithms. There are two outputs: cancer detection and tumor stages discrimination. In the first step, the AI-enhanced machine learning framework determines each signal as normal or cancerous, yielding a cancer likelihood score. In the second step, multiple classifier models, trained on cancer stages using the one-vs.-one strategy, generate metastasis evaluations of positive predictions from the prior step. We highlight the distinguishing performance of this system using 160 samples that included difference stage CRC patients (Figure 1B).

**Figure 2:**
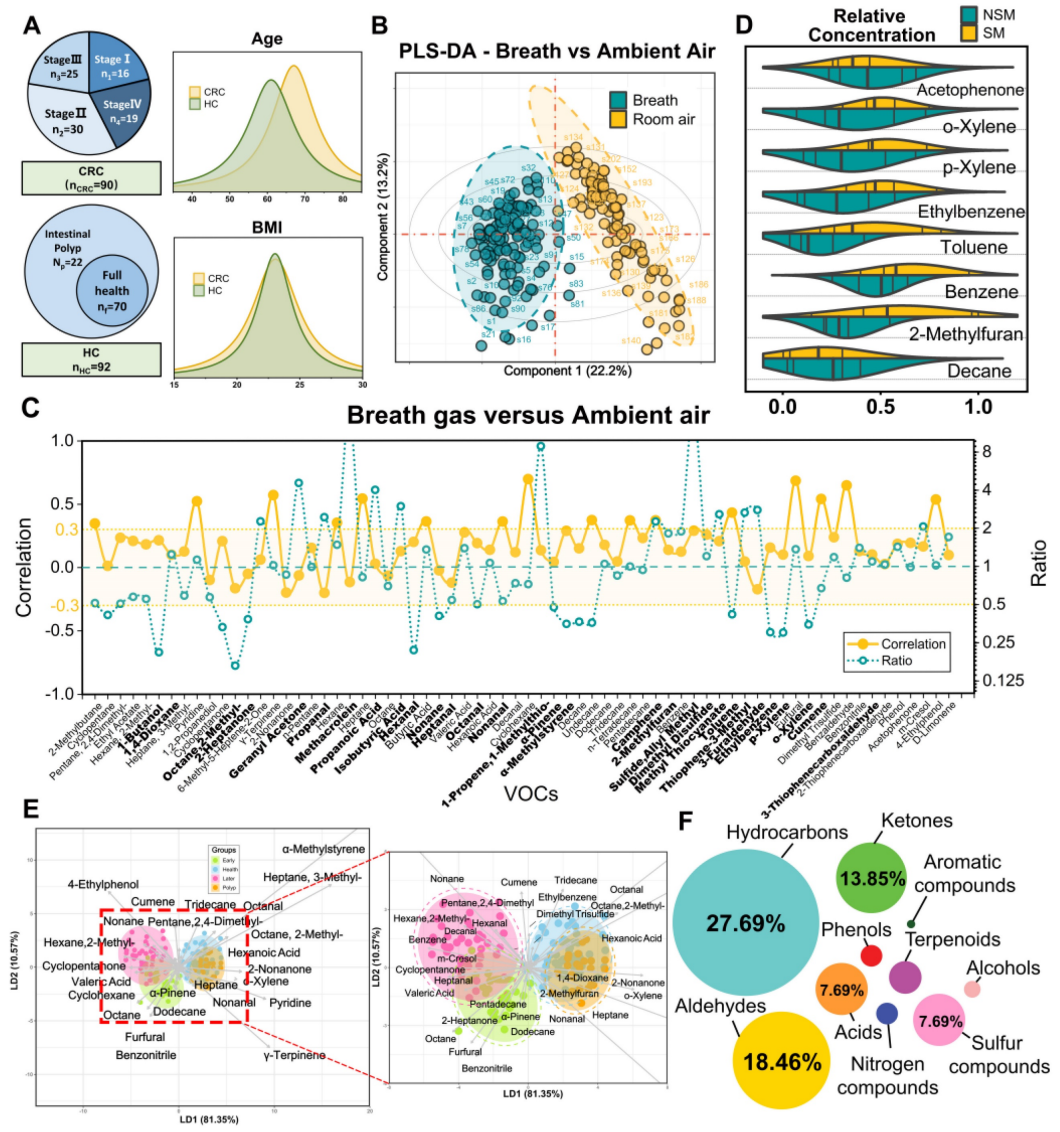
**Confounders exclusion and VOCs profile description. A.** Enrollment of the cohort study and BMI and age information of study participants. **B.** Breath and ambient air present distinct VOCs profiles. Supervised analysis with PLS-DA showed a clear separation between breath and ambient air VOCs profiles (R2Y = 0.90, Q2Y = 0.87, *P* < 0.05). Ellipses show 95% confidence intervals. **C.** Ratio of median from breath gas samples to median of ambient air samples and also the correlation of breath gas and ambient air samples. VOCs in bold are those with VIP greater than 1 in PLS-DA. **D.** The distribution of 8 smoking-related VOCs concentration between SM and NSM groups. **E.** Score plot of linear discriminate analysis (LDA) overview of breath VOCs among the healthy controls (HCs), Benign polyposis (polyps), CRC without metastases (early stage) and CRC with metastases (later stage) groups. **F.** The major chemical classes associated with CRC in this study and their percentage of candidate VOCs. Abbreviations: BMI, body mass index; SM, smokers; NSM, non-smokers.

A cohort of 194 participants, comprising 93 HCs and 101 CRC patients (including without metastasis and with varying degrees of metastasis), was recruited between July 2023 and November 2023. After excluding 12 individuals, there were 182 participants eligible for further analyses. The average age of the participants was 63.8 ± 16.8 years, with 56% being male. Both age and gender distributions showed balance between the HCs and CRC patients (*P* > 0.05), with the HC group being slightly younger (Figure 2A). Patients with CRC displayed a higher likelihood of having hypertension, elevated serum triglyceride levels, and increased insulin levels. Detailed baseline characteristics of these participants can be found in Table S1. The HCs contained some patients with mild intestinal polyps, which were analyzed by PCA and clustered heat maps with the completely healthy group in this study (Figure S1; Figure S2). It was found that there was almost no difference between the two groups, so we unified them together as HCs.

### Refined breath-VOCs profile minimizing confounding factors

Breath samples were gathered concurrently with corresponding ambient air at the Huadong hospital site and analyzed by GC-MS/MS. We detected and extracted a total of 72 VOCs from the chromatograms. Averaging was applied to repeated measurements before subjecting the normalized peak areas to principal component analysis (PCA) for outlier identification and removal. Using partial least squares—discriminant analysis (PLS-DA), we achieved a distinct separation between breath and ambient air samples (R2Y = 0.90, Q2Y = 0.87, *P* < 0.05) (Figure 2B). This separation was driven by 33 VOCs, with a variable importance projection (VIP) score > 1. A complete list of the VOCs characterizing each sample type and their respective VIP scores can be found in Table S2. This separation was also confirmed via low correlation between breath and ambient air (spearman r -0.3-0.3; Figure 2C), which exhibited our breath testing was independent from ambient air. Furthermore, Figure 2C shows the ratio of each VOC in human breath to ambient air. The median ratio is 1.28, indicating that the intensity of the breath VOC signal is usually higher than the corresponding ambient VOC signal, even though they are usually of the same order of magnitude.

We further examined the effects of smoking habits on breath VOCs derived from patients with CRC. The ANOVA and binary logistic analysis showed that eight smoking-related VOCs (benzene, toluene, ethylbenzene, o-xylene, p-xylene, acetophenone, 2-methylfuran, and decane) were independent risk factors for smoking habit, and should be excluded from subsequent analysis (Figure 2D; Table S3; Table S4). Moreover, we explored drinking habits as well as BMI and gender in the same way as above, but found no significant risk factors associated with these factors. Thus, the remaining 64 VOCs are listed in Table S5 as breath metabolites set after adjusting for these relevant confounders.

The breath-VOC profile of CRC was demonstrated by linear discriminant analysis (LDA) using breath metabolites set (Figure 2E). The first two principal components explained 96% of the overall variance. The CRC samples exhibited differentiable signatures compared to healthy control samples, as evidenced by their spatial separation in the LDA diagram, while polyps could not be discerned from the other HC samples. Similarly, the volcano plots revealed enrichment of these VOCs in the CRC cases. (Figure S3).

According to Metabolomics Standard Initiative level 1 criteria for metabolite identification, the most common chemical classes associated with CRC in this study included hydrocarbons (27.69%), aldehydes (18.46%), ketones (13.85%), acids (7.69%), sulfur compounds (7.69%), terpenoids (6.15%), alcohols (4.62%), phenols (4.62%), nitrogen compounds (3.08%) and aromatic compounds (1.54%) (Figure 2F). These volatile organic compounds exhibit multifaceted correlations, with short-chain fatty acids (SCFAs) presenting as self-associated clusters in both the heatmap and categorical correlation diagrams (Figure S4; Figure S5).

### Innovative AI-driven models for accurate CRC Diagnosis

**Figure 3:**
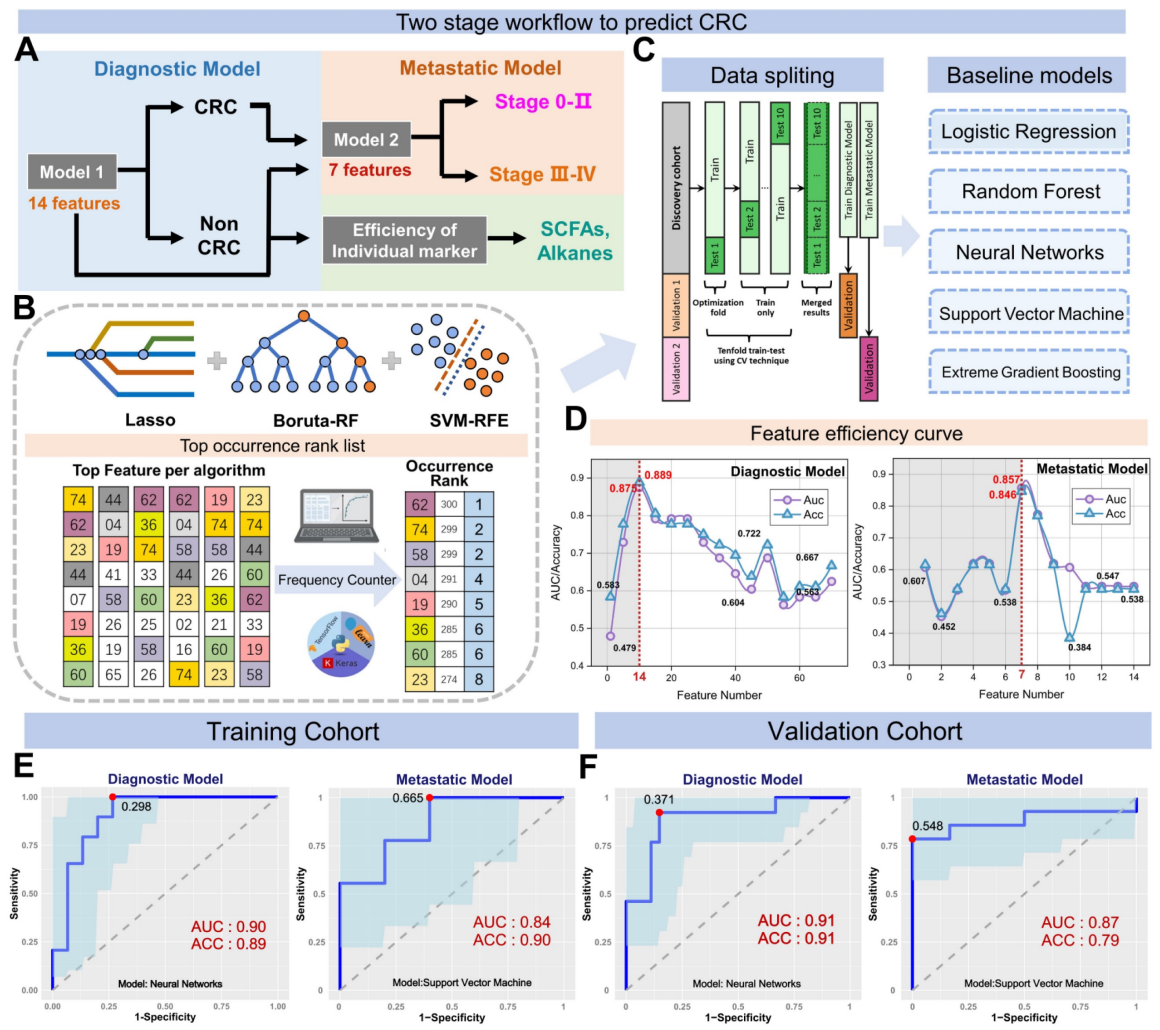
**Study flow chart, machine learning algorithms and their performance when using the two prediction models. A.** The two stages workflow for building the diagnostic and metastatic models with breath VOCs markers. **B-C.** The flow chart of integrating three algorithms' results in ranking features and integrating five classification algorithms in building classification models. **D.** The number of feature selection was determined by AUC and accuracy. **E-F.** The receiver operating characteristic (ROC) curves of Diagnostic Model and Metastatic Model that were used for predicting CRC and metastatic tumor in the training cohort (E) and the validation cohort (F).

We performed an AI-based study relying on breath VOCs set derived above to diagnose CRC with different stages. The study consisted of three parts: (1) AI-assisted feature importance list generation; (2) model-based variable selection; (3) model derivation and validation (Figure 3A). Breath samples were randomly assigned into training and validation cohorts with a 4:1 proportion.

In order to rank feature importance, we first built a selection frequency counter employing the SVM-RFE, LASSO and Boruta algorithms (Figure 3B; Methods for details). The frequency counter summarizes the selected features for each ML method during bootstrap procedure (Table S6). Following 100 iterations of the process, a feature importance list was generated by ranking total selection frequencies (Table S7). With the ranked list of breath VOCs, we constructed a comprehensive pipeline for variable selection. This process was accomplished by comparison of multiple baseline models, each of which displayed distinct adaptabilities to the original data structure upon the incremental input of 64 variables in descending order. These models included logistic regression (RL), random forests (RF), support vector machine (SVM), extreme gradient boosting (XGB), and neural networks (NNet), with corresponding mean AUCs of 0.71, 0.72, 0.74, 0.69, and 0.86 and mean accuracy of 72%, 75%, 77%, 75%, and 80%, respectively (Figure 3C; Table S8). In terms of its best discriminant performance, NNet was chosen to construct diagnostic model for CRC detection. Subsequently, NNet extracted the minimum 14 features from feature importance list by developing an efficiency sweet spot which was reflected in Fig 3d. These 14 features generated a diagnostic marker panel for CRC, comprising 3 short-chain fatty acids (SCFAs), 2 aldehydes, 2 ketones, 2 hydrocarbons, and 2 sulfur-containing compounds (Table S9).

By using a nested cross-validation approach, we finely tuned hyperparameters of NNet to fit this diagnostic marker panel, and finally constructed the Diagnostic Model. For CRC detection, the model achieved an AUC of 0.90, sensitivity of 89.1%, specificity of 89.6%, and accuracy of 91.6% in the training set, which consisted of 72 CRC and 74 HCs (n = 146) (Figure 3E). We also carried out a 10-fold internal cross-validation, yielding a verified AUC of 0.87 and an accuracy rate of 86.9%. Meanwhile, in the validation set comprised of 18 HCs and 18 CRC (n = 36), the Diagnostic Model attained 88.3% sensitivity, 92.3% specificity with an AUC of 0.91 (Figure 3E). These results indicated this model possesses outstanding performance for CRC detection and achieves great improvements compared to those models with only a single classifier in studies of Yang *et al.* 13.

Subsequently, we embarked on developing the Metastatic Model, which was designed to utilize the same methodology for detecting varying stages of CRC. After evaluating five baseline models, we determined that the SVM classifier yielded the most optimal results in terms of AUC and accuracy. Following the creation of the SVM feature efficiency curve, we were able to extract the top 7 features from a pool of 14 variables in the Diagnostic Model, as depicted in Figure 3D. These seven features encompassing 1 hydrocarbon, 2 aldehydes, 2 ketones, sulfide, allyl methyl and hexanoic acid, were defined as the metastatic marker panel (Table S10). Utilizing this panel, we optimized the hyperparameters of SVM and developed the final Metastatic Model. This model demonstrated a sensitivity of 81.1%, a specificity of 84.0%, an accuracy of 87.2%, and an AUC of 0.87 when distinguishing CRC patients with and without metastases (Figure 3F). While a better result was acquired for identifying NM from the metastatic subgroups LNM and DM, yielding an identification accuracy of 82.4% and 89.6%. To further examine the sensitivity of Metastatic Model, we evaluated the performance of Diagnostic Model in discerning LNM and DM from NM group. These paired comparisons generated AUCs of 0.500 and 0.638, respectively (Figure S6), indicating the Diagnostic Model has limited predictive power for metastatic stages. The comparative analysis of two models highlights the better sensitivity and reliable performance of the Metastatic Model with 7 features in distinguishing CRC with metastasis. Overall, our Diagnostic/Metastatic models exhibit excellent at identifying both CRC and different staging types, and provide a powerful complement to existing CRC diagnostic techniques.

**Figure 4:**
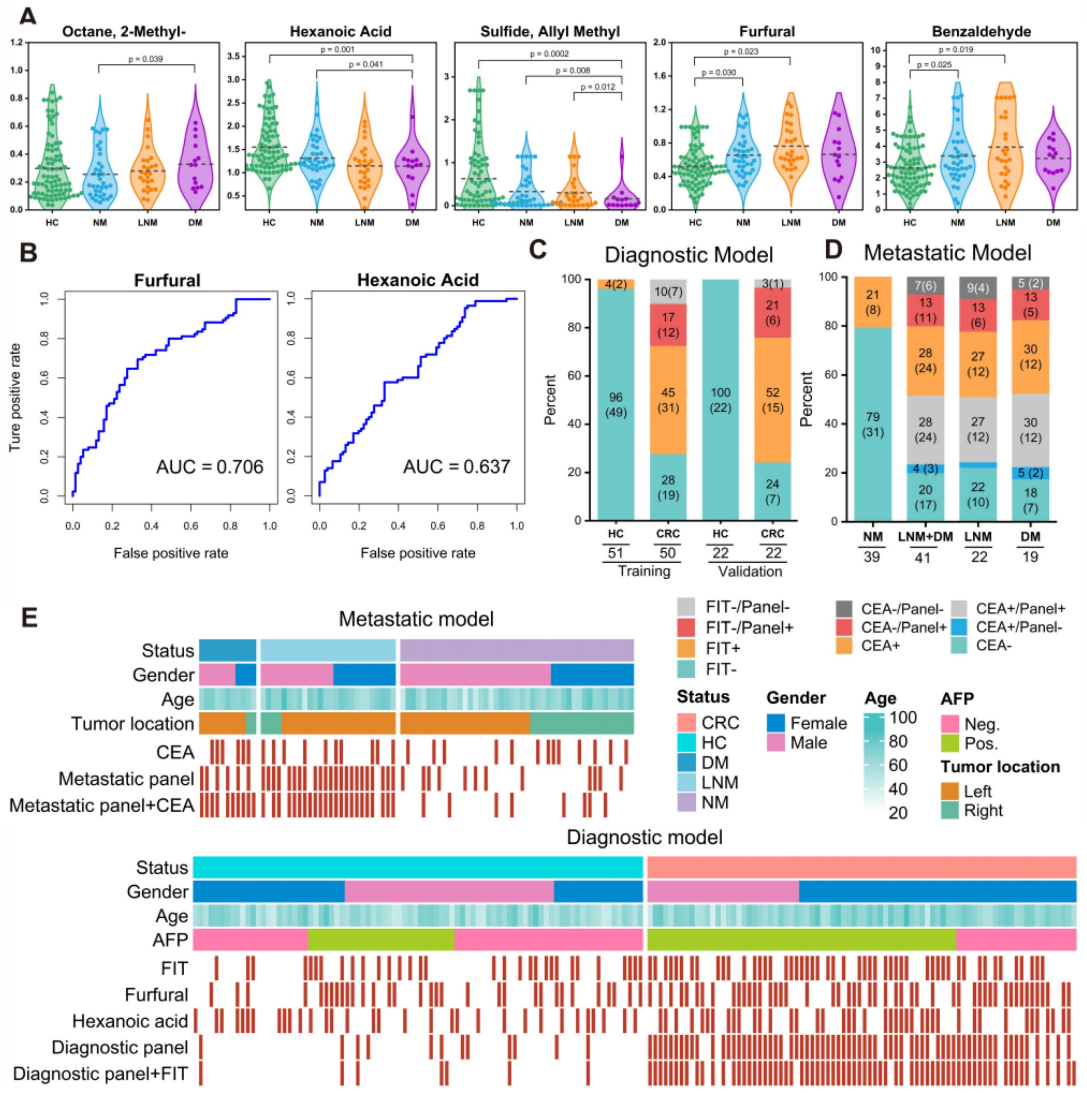
**Verification of the breath biomarkers using comparative analysis. A.** Scatter plot for octane, 2-methyl-, hexanoic acid, furfural, sulfide allyl methyl, and benzaldehyde in 92 healthy controls (HCs) and 90 CRC patients, including CRC without metastases (NM; n = 46), CRC with lymph node metastasis (LNM; n = 25) and CRC with distant metastasis (DM; n = 19). The median values in each group are shown as black dotted lines. The differences between groups for each marker were analyzed by two-sided Kruskal-Walli's test. **B**. The independent diagnosis efficiency of two key markers among the fifteen markers in the diagnostic model. **C**. ROC curve of serum CEA, metastatic marker panel, and the combination of the metastatic panel and CEA for the metastatic model (LNM+DM vs. NM). **D**. Diagnostic and metastatic predictive power of the diagnostic markers and metastatic markers in the individuals who were misdiagnosed by the FIT test or serum CEA. The values in parentheses indicate the number of samples corresponding to each percent. +, positive; -, negative; n, number of samples. **E**. Heatmap of the dot plot data for single breath markers as well as the diagnostic or metastatic panel with a specificity of 95%, and the combination of corresponding clinical biomarker indices for the diagnostic or metastatic model was considered positive when either the panel or FIT/CEA was positive. Red: positive using the cutoff value with a specificity of 95%. The FIT test, serum CEA, tumor location, sex, and age are indicated by color-coding. *CRC* colorectal cancer, *FIT* fecal immunochemical test, *CEA* carcinoembryonic antigen, *AFP* alpha fetoprotein, *Neg.* negative; *Pos.* positive; *NA* not available.

We further assessed the efficiency of 14 markers in the diagnostic model (Figure S8) and found that furfural and hexanoic acid showed the most favorable performance with AUCs of 0.706 and 0.637 in the training cohort. (Figure 4B). Additionally, metastatic markers furfural and Octane-2-methyl were significantly higher in CRC patients' breath, while hexanoic acid, sulfide, and allyl methyl levels decreased with cancer progression, reaching the lowest levels in DM (Figure 4A). These findings provide valuable insights into potential markers for metastasis and indicate their association with CRC advancement.

### Breath VOCs biomarkers complemented FIT and serum CEA

For comparison, we concurrently assessed the fecal immunochemical test (FIT), a recognized CRC screening biomarker, in 145 fecal samples. These were methodically distributed into two datasets: the training set, comprising of healthy controls (HC), n = 51, and CRC patients, n = 50; and the validation set with HC, n = 22, and CRC, n = 22. In the training cohort, FIT demonstrated a sensitivity of 62.8% (31/50) and an impeccable specificity of 95.8% (49/51). Contrarily, within the validation cohort, the sensitivity was recorded at 70.2% (15/22) while maintaining a specificity of 100% (22/22). Concurrently, our novel breath diagnostic marker panel exhibited a commendable area under the curve (AUC) of 0.902 with a sensitivity of 85.7% and specificity of 86.3% in the training set. In the validation set, the figures stood at 0.910, 88.7%, and 91.6%, respectively. Notably, the diagnostic sensitivity proffered by the breath marker panel surpasses that of FIT. Analyzing both the training and validation cohorts, the breath marker panel augmented diagnostic precision for an additional 12 patients (24.0%) and 6 patients (27.2%), respectively, as visualized in Figure 4C. Of those that returned negative results via FIT in CRC, the training set accurately diagnosed 63.6% (12/19), and the validation set achieved an impressive 87.5% (6/7) through the breath diagnostic marker panel. The combination of both FIT and the breath diagnostic marker panel heralds a more proficient diagnostic approach, manifesting a sensitivity and specificity of 88.8% and 94.1% in the training set, with corresponding figures of 91.8% and 92.4% in the validation set.

Shifting our focus to the metastatic dimension, the widely acknowledged clinical CRC metastasis biomarker, CEA, was carefully evaluated in 80 serum samples. The overarching results suggest that the breath metastatic model's efficacy parallels that of serum CEA. When we integrated the breath VOCs marker panel with serum CEA, there was a significant increase in predictive efficacy compared to the standalone use of CEA. This combination manifested an AUC of 0.939, with sensitivity, specificity, and accuracy metrics at 90.9%, 88.2%, and 93.5%, respectively. Adopting the established clinical CEA threshold of 5 ng/mL to distinguish metastatic from non-metastatic CRC, serum CEA unveiled a sensitivity of 58.1%, a specificity of 78.0%, and an AUC of 0.615 (Figure S7). Within the cohort diagnosed with metastatic CRC, CEA identified a total of 24 individuals (58.5%). The breath metastatic markers further augmented the diagnostic power by recognizing an additional 10 patients (24.4%). Subgroup analyses elucidated the metastatic model's enhanced discriminatory capacities, identifying an extra 6 patients (27.2%) with lymph node metastasis (LNM) and 5 patients (26.3%) with distant metastasis (DM), compared to CEA's detection of 12 individuals (54.5%) with LNM and 12 cases (63.2%) with DM (Figure 4D). Of the cohort who were CEA-negative in LNM or DM categorizations, 60.0% (6 out of 10) and 71.4% (5 out of 7) were accurately identified as having metastases via our marker panel.

To better understand the effectiveness of breath VOCs in detecting CRC and determining the risk of metastasis, we utilized the cutoff values of each key VOC and classifiers of each model on both training and validation cohorts. When it comes to diagnosis, combining the marker panel with the FIT showed a sensitivity of 88.8% and specificity of 94.1% in the training set, and 91.8% and 92.4% in the validation set, respectively (Figure 4E- bottom). At the same time, when it comes to assessing metastatic risk, using the marker panel along with serum CEA resulted in a sensitivity of 90.9% and specificity of 88.2% (Figure 4E-upper). It's worth noting that the marker panel consistently outperformed individual compounds in terms of sensitivity. Moreover, when combined with FIT or serum CEA, the marker panels from both approaches significantly improved diagnostic accuracy.

### Tracing CRC breath markers to gut microbiota origins

**Figure 5:**
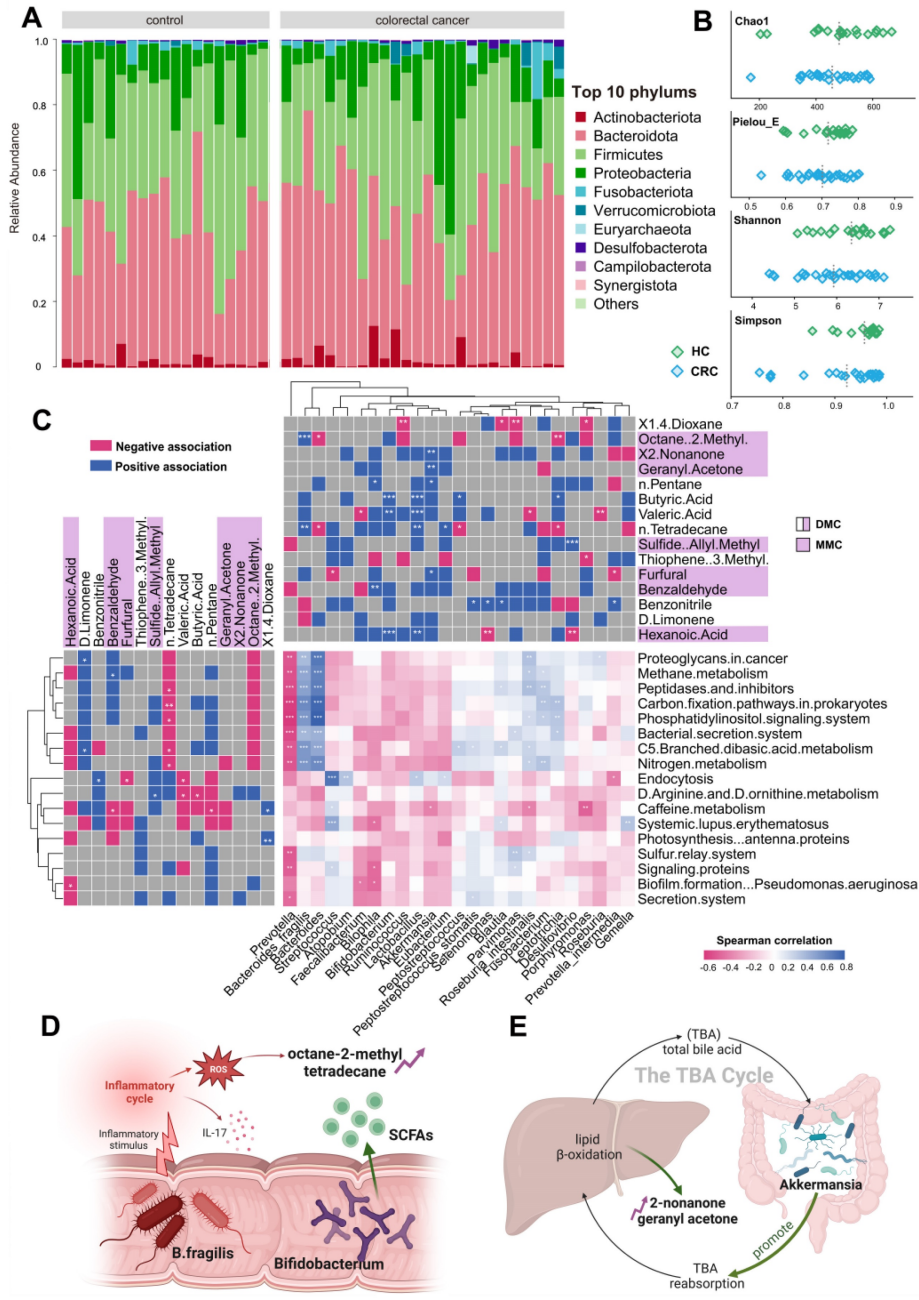
**Combined analysis of gut microbiome and breath VOCs. A.** Component proportion of bacterial phylum in each group; n = 25 for the CRC group and n = 19 for the HC group. **B.** The alpha diversity. **C.** The tripartite correlation heatmap of gut microbial species in CRC, KEGG pathways modules and breath markers. The left panel denotes the Spearman correlations between pathway modules and breath markers. The top panel denotes the Spearman correlations between species and breath markers. **D.** Metabolic pathways of alkane-based markers in relation to inflammatory factors and reactive oxygen species and sources of bacterial gas production for SCFAs markers. **E.** Relationship of ketone markers metabolic pathways to the hepatic and intestinal circulation. *DMC* Diagnostic Marker of CRC, *MMC* Metastatic Marker of CRC.

The gut microbiome and its metabolites play roles in both CRC development and progression. Our study utilized 16S rDNA sequencing techniques to shed light on the origins of exhaled CRC biomarkers, revealing potential associations with gut microbiota. For this purpose, we collected fecal samples from 44 participants, including 25 CRC patients and 19 HCs. Initially, we enumerated the top 10 species in terms of abundance at the phylum levels for the two groups and depicted these in relative abundance bar charts (Figure 5A). Upon aligning sequences to assess bacterial diversity differences, we noted significant variations in the Shannon (6.03 ± 1.39 vs. 6.55 ± 0.90, *P* = 0.053) and Chao1 indexes (425.70 ± 79.63 vs. 485.81 ± 127.98, *P* = 0.048) between CRC and HC groups (Figure 5B). Weighted and Unweighted PCoA plots illustrated group segregation based on the first three PCoAs (Figure S9). These findings imply that the richness and diversity of gut microbiota could be significantly shaped by the tumor burden, providing an analytical basis for exploring the metabolic pathways related to CRC exhaled biomarkers.

Based on the PICRUSt2 function prediction of the 16S rDNA sequence, 17 functional pathways of significantly different were observed between CRC group and HCs group (Data S1; Figure S10). Notably, among 17 functions pathways, only five were upregulated in CRC (log2 (Control/CRC) < 0, *P* < 0.05, FDR < 0.05), all of which are involved in energy utilization, cell signaling, and host interactions. Next, we plotted a tripartite correlation heatmap using the breath biomarker data 14, PICRUSt2 functional enrichment data and species abundance data (Figure 5C).

A total of 12 VOCs exhibited significant associations with 15 metabolic pathways and 21 prominently enriched species (*P* < 0.05). This underscores the pivotal role of microbial activities and taxa in interactions with breath VOCs in influencing host well-being. Significantly positively correlations were detected between B. fragilis and those five upregulated pathways (Figure 5C, blue module; r > 0.7, *P* < 0.01). This suggests that B. fragilis of CRC patients may not only enhance energy substrate utilization efficiency but also intensify host inflammatory responses and possibly promote the spread of cancer cells. Such insights indicate close attention to the impact and role of B. fragilis in CRC progression.

As evidenced by Figure 5C, exhaled dimethyl octane and tetradecane positively correlate with B. fragilis (Bacteroides), while D-limonene negatively correlates. Dimethyl octane and tetradecane are common markers of oxidative stress in human, contrast with antioxidant D-limonene. Concurrently, supported by studies from Du and Bhandari, B. fragilis is widely associated with inflammatory responses that can promote cancer cell proliferation by triggering the IL-17 inflammatory cascade response 15-17. This association elucidates a possible mechanism of tumor proliferation driven by B. fragilis that augments the production of dimethyl octane and tetradecane while inhibiting D-limonene (Figure 5D). Furthermore, Figure 5C reveals positive correlations between exhaled 2-nonanone and geranyl acetone and Akkermansia. These ketones are produced during lipid β-oxidation and linked to activities in liver. Akkermansia stimulates their production by enhancing hepatic and intestinal circulation of TBA, thereby exhibiting anti-inflammatory and anti-cancer effects. This observation supports the notion that elevated concentration of 2-nonanone and geranyl acetone in exhaled breath of CRC may be a consequence of Akkermansia's positive regulation on lipolysis (Figure 5E). Significant reductions in exhaled butyric and valeric acids in CRC were observed, corresponding with decreased abundances of Bifidobacterium and Lactobacillus. These bacteria have been implicated as SCFAs-producers in several studies. In this way, they serve as the primary energy source for colorectum epithelial cells and exert a positive effect on gut health. This reduction in butyric and valeric acids is most likely due to declines in beneficial acid-producing bacterial abundance in gut (Figure 5D). Additionally, an increased exhaled allyl methyl sulfide concentration associating with up-regulation of Desulfovibrio was noted in the CRC group (Figure 5C). Desulfovibrio metabolizes thioethers into thiols via sulfur reductase, while producing hydrogen sulfide. This toxic gas potentially damages intestinal mucosal barriers, induces inflammatory responses, and eventually contributes to the development of colorectal cancer. Therefore, the elevation in exhaled allyl methyl sulfide concentration may originate from the dominant proliferation of Desulfovibrio.

Through our investigations, production mechanisms of most substances in the breath marker panels have been identified through relevance studies with the gut microbiota, which related to inflammatory response, lipid oxidation, energy supply, and cellular damage. Our findings suggest that gut microbiota activities contribute to understanding the generation mechanisms of exhaled VOCs in CRC patients, underscoring the feasibility of exhaled markers in clinical diagnosis and metastasis prediction for CRC.

## Discussion

This study focuses on the early diagnosis of colorectal cancer through an extensive investigation of breath markers. Utilizing TD-GC×GC-QQQ-MS technology, we successfully identified a total of 72 VOCs from the breath samples of 90 colorectal cancer patients and 92 healthy controls. After adjusting for confounding factors, advanced AI methods were subsequently employed to construct both the CRC Diagnostic and Metastatic Models. These models revealed the presence of fourteen diagnostic markers and seven metastatic markers, which showcased superior performance compared to conventional tests like CEA and FIT. Moreover, through a comprehensive analysis involving the gut microbiome, we established a connection between these markers and inflammatory responses, lipid metabolism, and other significant factors. This sheds light on their potential not only as indicators of colorectal cancer dynamics but also for fostering advancements in clinical applications.

The utilization of exhaled VOCs for diagnostic purposes in CRC faces limitations due to confounding factors. In order to mitigate these limitations, we implemented a system that utilizes TD tubes with the ReCIVA sampler for breath sample collection. This method of sampling provides clean air and is superior to traditional equipment such as airbags, effectively limiting interference from ambient air and ensuring accuracy of respiratory data. Monitoring pressure and CO_2_ levels during patient breathing in real-time proved to be essential to achieving accurate capture of breath samples. Our correlation analysis revealed minimal influence from ambient VOCs, indicating that our method successfully mitigates ambient VOC interference. These findings are consistent with Di Gilio's comparative study on respiratory sampling techniques 18. Previous research has shown that physiological and habitual factors such as age, BMI, smoking, and drinking may impact the distribution of VOCs in exhaled breath. However, our variance and regression analyses did not identify age, BMI, and drinking as significant risk factors for CRC. Smoking, however, was found to significantly relate to five BTEX compounds (benzene, toluene, ethylbenzene, and xylene) and 2-methylfuran in human breath. BTEX compounds have been previously identified as secondary products of cigarette combustion due to their positive correlation with exhaled CO levels 19. 2-methylfuran has also been found to be an effective indicator for identifying smoking participants, as demonstrated by Alonso *et al.* 20. Consequently, we excluded these smoking-related VOCs from further investigation. These results highlight the effectiveness of our approach in minimizing potential confounders and maintaining the integrity of subsequent screening programs involving exhaled biomarkers.

Subsequently, we developed an AI-based model to identify breath markers for CRC detection. During the modeling stage, we integrated the outcomes obtained from three AI-driven feature selection techniques: the RFECV algorithm utilizing recursive action, the Boruta algorithm employing random shadow generation, and the LASSO algorithm employing penalized shrinkage training. This synergy of techniques within our approach addressed issues of feature redundancy and selection bias that have been observed in previous model-based biomarker studies 21, which often neglected the crucial step of comprehensive feature selection or relied solely on a singular method 22. Moreover, our comparison between models with and without feature ranking revealed a substantial disparity in performance (AUC 0.76-0.95 vs 0.46-0.70), thereby validating the efficacy of our feature selection outcomes. This phenomenon can be attributed to the successful elimination of noise features utilizing the sequential inputting strategy (Figure S11). In the event that all features were incorporated into the models, there would be a heightened risk of overfitting and diminished performance, as emphasized by Wang *et al.* in their study 23, 24. Furthermore, by utilizing a feature importance list, we assessed the adaptability of five baseline classifiers in terms of mean AUC and accuracy. We then selected the most optimal classifier among them for subsequent modeling processes. When compared to prior studies on biomarker modeling conducted by Halner *et al.* 25, 26, the classification accuracies achieved by our models exhibited significant enhancements. This improvement can likely be attributed to our meticulous consideration of the compatibility between classifiers and data structures. Overall, our AI methodology assures precise biomarker identification, enhances the utility of models, and provides a reliable means for non-invasive CRC diagnosis through exhalation.

In utilizing our AI-driven methodology, we identified breath markers for CRC comprising 3 SCFAs, 3 aldehydes, 5 hydrocarbons, and 3 sulfur-containing compounds. We then evaluated and compared the performance of these breath markers with that of FIT or CEA, which are the primary globally recognized CRC-specific tests used by physicians and patients 27. Our Diagnostic Model, based on these breath markers, enhances sensitivity by almost a third compared to FIT. Additionally, Remarkably, 24% of the FIT-negative CRC patients in the training cohort and 27.2% in the validation cohort were correctly identified through our diagnostic model. When evaluating tumor invasion and metastasis, the Metastatic Model outperforms CEA in accuracy, increasing it from 69.2% to 93.5% (+24.3%). In comparison to relying solely on CEA (≥5 ng/mL), incorporating CEA with the metastatic markers increases sensitivity from 58.1% to 90.9%, representing a significant improvement of 32.8% for all metastatic patients. These analyses reveal that our exhaled markers not only capture physiological alterations in cancer patients but also discern CRC with greater accuracy and efficiency. Additional further research is needed to investigate if our breath metastatic markers can serve as an early warning for recurrent CRC.

We undertook an exploration of sources and production processes of respiratory biomarkers that can be utilized for CRC detection. Numerous studies have pointed towards the significant role of the gut microbiome in CRC tumorigenesis and progression, potentially via microbial metabolites, triggering pro-inflammatory responses, and affecting energy equilibrium within cancer cells 28. With this understanding, we combined fecal bacterial 16S rDNA sequencing results with breath markers to elucidate potential associations. Consistent with our findings, existing literature indicates an elevation in the levels of alkanes and methylated alkanes in the exhalations of cancer-afflicted individuals 29. The origins of these methylated alkanes remain a subject of contention; however, prevailing sentiments within the academic community suggest that they are byproducts of oxidative stress 30. Our results support this perspective, revealing that the pro-inflammatory role of B. fragilis intensifies oxidative stress *in vivo*, culminating in heightened levels of dimethyl octane and tetradecane in the breath of CRC patients. It is noteworthy that ketones, closely connected to augmented fatty acid oxidation in various cancers 31, are expected to largely stem from gut microbiome dysfunction 32. This observation dovetails with our discovery regarding Akkermansia's facilitative role in lipolysis. Interestingly, a study by DeBerardinis *et al.* has found that the lipid membrane of cancerous cells exhibits a pronounced saturation compared to their benign counterparts 33. This lipidic interplay could potentially explain the elevated aldehyde concentrations in the breath of CRC patients 34. While the precise origins of benzonitrile and 3-methylthiophene in our marker panel remain unclear, previous studies have attributed them to food or industrial sources 35, 36. In summary, the variations in our acid, ketone, and hydrocarbon breath markers primarily result from imbalances in gut microbiome, while aldehydes are influenced by changes in the cellular microenvironment. These intertwining influences highlight the intricate relationship between the gut microbiome and CRC progression and warrant further exploration in future studies.

Meng *et al.* applied HPPI-TOFMS to study the breath test of cancer patients, and they used a Tedlar gas bag to collect the patients' breath gas 37. The samples were collected one breath at a time due to the bag's capacity limitation, and the entire collection process lasted only 60 seconds. This presents a significant issue, as breath markers with low concentrations may not reach the detection limit and therefore cannot be detected by the instrument. In addition, our improved breath sampler uses TD adsorbent tubes to concentrate and collect the subject's breath for 15-20 minutes, with a volume of up to 2L. This eliminates the risk of exogenous pollutants and loss of VOCs during storage. Furthermore, the machine learning component of our system exclusively employs SVM algorithms to build the model. While this approach has limitations, we have determined it to be the most effective modeling tool for our purposes. It appears that this study did not have a feature screening process, but rather included all detected substances in the training. As a result, the bio-interpretability of the findings was poor, and they subsequently failed to identify a breath marker for lung cancer. In contrast, our platform integrates multiple machine learning algorithms to form a classifier evaluation pipeline that identifies the optimal solution for the classification algorithm based on experiments with a high-capacity collection of samples. Fourteen VOCs were identified as diagnostic markers for colorectal cancer. The biological origin of these markers was also discussed from the perspective of gut microbiome. Altomare *et al.* used a similar breath sampler for CRC-related study, but also suffered from insufficient sampling time and oversimplified method for screening markers 38. Their results showed that ethylbenzene and methylbenzene were key VOCs for colorectal cancer, but these two substances have been identified as exogenous in several studies, and they were found to be smoking-related confounders and were excluded in our study. In conclusion, our platform demonstrated greater rationality and superiority in sample collection and analysis, data cleaning, classification modeling, and source interpretability compared to similar work.

Limitations of this study should be considered when explaining these results. Firstly, the restricted sample size of advanced CRC patients may compromise the precision of the Metastatic Model in differentiating between stage III-IV cancer participants. Therefore, further validation is necessary to ensure accuracy. Secondly, all breath samples used for this study were collected solely from Huadong Hospital, which could introduce bias due to the absence of multi-center external validation. Thirdly, we focused solely on the basic classification labels of CRC, without digging into the details of subtype categorization 39. Additionally, the relationship between our breath markers and the gut microbiome during CRC progression remains unclear. This requires multiple follow-up samples from key patients, which we plan to implement in future studies. Research on breath biomarkers is still in the exploratory phase, and the methods used are relatively complex. This currently limits large-scale clinical applications. The ultimate goal of this research is to develop a simple and inexpensive portable device that can provide results as quickly as an alcohol test, thus achieving good results in disease screening.

Despite these limitations, our work offers promising results for non-invasive CRC diagnosis. Our investigation identified potential associations between breath biomarkers and the gut microbiome, revealing possible metabolic mechanisms underlying these biomarkers. Ultimately, our findings present exciting innovations for reliable CRC detection and offer insight into potential metabolic approaches for treating the disease.

## Methods

### Study participants

A total of 90 eligible CRC patients (50 males and 40 females; median age 67 ± 17.1 years) were recruited from Huadong Hospital affiliated to Fudan University in Shanghai, from July 2023 to November 2023. All cohorts were recruited simultaneously and consecutively throughout the study. CRC diagnoses were confirmed through histological examination of tissues and radiological imaging, and breath samples were obtained in the morning before any surgical, chemotherapeutic, or radiotherapeutic intervention. Patients who had recovered from surgery accounted for 6.7% of exclusions, as well as those with alternate pathological diagnoses such as mucinous adenocarcinoma, melanoma, and other non-CRC tumors. Following these criteria, 90 patients remained eligible, and were categorized based on the absence or presence of metastasis into three groups: 46 without metastasis (NM), an incorrectly cited number for those 25 with local node metastasis (LNM), and 19 with distant metastasis (DM). The NM group was defined as early stage, while the LNM and DM groups were classified as advanced stage based on the presence of metastatic lesion in the tumor. The CRC staging utilized the TNM system endorsed by the Union for International Cancer Control (UICC).

Simultaneously, Huadong Hospital recruited 92 healthy controls (HCs) with a median age of 61 years (range: 22-83 years), including 53 males and 39 females. Among them, 23 individuals were diagnosed with mild intestinal polyps, while the remaining 65 were deemed completely healthy. These individuals typically underwent a comprehensive physical examination, including colonoscopy and gastroscopy, during their 2 to 3 day hospital admission. They were selected based on criteria including no history of tumors, a clean bill of health from a physical exam, and no respiratory diseases in their medical history. Table S1 presents a comparison of the demographic and clinical data of the 182 CRC patients and HCs. A schematic diagram of participant recruitment and sample allocation proportions for model construction is provided in Figure S12. All participants entered the study with informed consent. The research adhered to the principles of the Declaration of Helsinki and received approval from the Ethical Committee at Huadong Hospital (KY 2023K127).

### Breath sampling methodology

Once obtaining informed consent from all patients, we strictly followed a standardized sampling procedure using an enhanced sampler comprised of breath biopsy cartridges and a porTable air supply for exhaled sample collection. The Method S1 provides a description of the parameter optimization scheme and detailed internal structure of the improved breath sampler. To minimize the interference of confounding factors, we performed sample collection between 7:00 and 8:00 am after an overnight fast. Patients were also asked to rest in the same area for at least 20 minutes before sampling. For each participant, we collected 2L of alveolar breath gas with corresponding ambient samples. Target VOCs were collected in two duplicate multi-layer thermal desorption (TD) tubes containing Carbograph 5 TD and Tenax/TA (Markes biomonitoring tubes, Markes International Ltd, UK).

### Pretreatment and instrumental analysis

Following quality control measures on the samples, the TD tubes were analyzed using a comprehensive mass spectrometry-based procedure composed of TD-GC-MS/MS. The thermal desorption instrument (TD, from Marks Company, UK) first pre-purged the TD tubes for 10 minutes at a helium flow rate of 100 mL/min to remove moisture and oxygen from the samples. The TD tubes were then heated to 300 °C for 5 minutes to desorb the samples, and the desorbed VOCs were concentrated in an internal focusing cold trap at 30 °C. After purging the focusing cold trap with helium gas at a flow rate of 25 mL/min for 2 minutes, it was rapidly heated to 300 °C and maintained for 5 minutes. During the heating process, VOCs were desorbed from the focusing cold trap and injected into an Agilent 7890A gas chromatograph coupled with an Agilent 7000B triple quadruple mass spectrometer (GC-MS/MS, Agilent Technologies Inc., USA) through a 180 °C transfer line in a non-split mode for qualitative and quantitative analysis of VOCs. The GC employed a J&W Scientific DB-624 chromatographic column (60 m, internal diameter 0.25 mm, film thickness 1.4 μm), with an injection port temperature of 250 °C. The oven was maintained at 40 °C for 5 minutes, then ramped at 5 °C/min to 160 °C, followed by a 10 °C/min ramp to 230 °C, where it was held for 21 minutes. The ion source and MS transfer line temperatures were set at 230 °C and 250 °C, respectively. The MS was operated in full-scan mode for analyte identification, with a mass range (m/z) of 30-350. Quantitative analysis was performed in Selected Ion Monitoring (SIM) and Multiple Reaction Monitoring (MRM) modes. The chemical characteristics of each peak were confirmed by reference to the National Institute of Standards and Technology (NIST) mass spectral library (version 2.3). After confirming the retention time and mass spectrum of the target compounds in SCAN mode, quantitative analysis was performed in Selected Ion Monitoring (SIM) and Multiple Reaction Monitoring (MRM) modes. The Agilent MassHunter quantitative analysis software and the Agile2 integrator were used to automatically integrate compound peaks, with manual adjustments made as necessary. A combination of external standard curves and internal standard normalization was used to quantify 82 VOCs.

### Potential Confounding Evaluating methods

To gauge the impact of environmental air on human breath, we constructed a Partial Least Squares Discriminant Analysis (PLS-DA) and computed z-scores. The significance of PLS-DA models was assessed using the 'ropls' package. We deemed compounds with a variable importance in projection (VIP) score exceeding 1 as significant for classification purposes. Additionally, we analyzed PLS-DA model loadings to ascertain the contributions of different groups. The Wilcoxon rank-sum test was employed for univariate analyses, with the Benjamini-Hochberg method correcting for false discovery rates. Normal distribution was not characteristic of most volatile organic compounds (VOCs); thus, we employed the two-tailed Mann-Whitney U test for detecting significant disparities across datasets. This nonparametric test ranks individual values collectively from both datasets and is as robust as the standard Student's t-test for identifying shifts in median values, without the requirement for normal distribution. A z-score magnitude greater than 1.96 typically signifies a statistically meaningful difference between two datasets at the 5% significance level 40.

We handled physiological and habitual confounders by applying ANOVA and binary logistic regression to eliminate significant risk factors. Given the smaller sample size and non-normal data distribution, we compared breath VOC concentrations between smokers and non-smokers using a one-way ANOVA for preliminary p-values, considering p < 0.05 significant. This aided in selecting potential smoking-related VOC candidates. Subsequently, binary logistic regression models were formulated to evaluate the potential of these VOCs in association with smoking in CRC patients. We plotted Receiver Operating Characteristic (ROC) curves and computed the areas under the curves (AUCs) to appraise the diagnostic accuracy of these risk factors, with a p-value less than 0.05 in a two-tailed test indicating statistical significance.

### AI-assisted discovery of candidate breath biomarkers

Training and testing of the models were executed using R Version 4.2.1, utilizing a suite of packages including random Forest, e1071, glmnet, rpart, caret, xgboost, and cvAUC for machine learning tasks 41. We developed two separate analytical frameworks: one aimed at distinguishing CRC patients from healthy individuals (the Diagnostic Model) using breath VOCs signatures, and another (the Metastatic Model) for differentiating between early and advanced stages of cancer in those diagnosed with CRC.

Both models underwent a consistent two-stage construction process, initiated by an AI-driven feature ranking executed through three advanced machine learning techniques: (1) a variant of the linear support vector machine recursive feature elimination (SVM-RFE) algorithm 42, (2) Least Absolute and Shrinkage and Selection Operator (LASSO) with L1 penalty and embedded feature selection, and (3) Boruta package characterized by shuffling shadow features and binomial distribution conception. Subsequent procedures involved an 80:20 train-to-validation dataset division, where breath VOC signatures underwent scrutiny based on aggregated selection counts from 100 bootstrapped random samples across the three evaluative methods. This rigorous analysis culminated in the generation of comprehensive feature importance hierarchies for each model (Figure 3B).

We refined our methodology by employing five baseline models—LR, RF, SVM, NNet, and XGB. These models were tasked with pinpointing the least number of features necessary to maximize the AUC and accuracy. Selection of the ultimate classifier depended on its superior average performance metrics during training iterations, a process that enabled the isolation of vital features for accurate CRC diagnosis. To construct a robust model, we implemented 10-fold cross-validation (with a training-to-test data ratio of 90:10) using the most effective classifier identified from the initial models. The validation cohort was used to valid our training model to avoid overfitting. During the model construction process, 182 eligible breath samples were randomly assigned to the training and test sets in an 8:2 ratio. The AUC was estimated using ROC analysis from the pROC package to evaluate model performance. An optimal probability threshold was derived based on the maximum Youden index of the model (sensitivity + specificity - 1). Samples with values below or above the critical value will be predicted as healthy controls and colorectal cancer, respectively.

### 16s rDNA sequencing experimental procedure

DNA extraction from fecal samples was performed utilizing the TianGen Magnetic Soil and Stool DNA Kit (TianGen, China, Catalog #: DP712). Various regions of the 16S rRNA/18SrRNA/ITS genes (e.g., 16SV4/16SV3-V4/16SV4-V5, 18SV4/18SV9, ITS1/ITS2, ArcV4) were amplified using primers specific to each region (for instance, 16SV4: 515F-806R, 18SV4: 528F-706R, 18SV9: 1380F-1510R) including barcodes for identification. The amplification process involved 15 µL of Phusion® High-Fidelity PCR Master Mix (New England Biolabs), 0.2 µM of each primer, and approximately 10 ng of template DNA. The PCR protocol started with a 98°C denaturation step for one minute, followed by 30 cycles at 98°C for 10 seconds, 50°C for 30 seconds, 72°C for 30 seconds, and a final extension at 72°C for five minutes. Post-amplification, PCR products were combined with a loading buffer containing SYB green and subjected to electrophoresis on a 2% agarose gel for verification. Equal-density PCR products were pooled and purified using TianGen's Universal DNA Purification Kit (Catalog #: DP214). Sequencing libraries were prepared with the NEB Next® Ultra™ II FS DNA Library Prep Kit (Catalog #: E7430L), according to the manufacturer's instructions, and assessed via Qubit, real-time PCR, and bioanalyzer analyses for quantification and size distribution.

For sequence processing, barcodes and primer sequences were trimmed from the paired-end reads, which were then merged using FLASH (V1.2.11). FLASH is renowned for its speed and precision in overlapping paired-end reads from the same DNA fragments. The resulting raw tags underwent quality filtering with fastp (Version 0.23.1) to yield high-quality clean tags. These tags were then screened against the reference Silva database (for 16S/18S) or Unite Database (for ITS) using the UCHIME algorithm to remove chimeric sequences, leaving us with effective tags. Further denoising was done using DADA2 or the deblur tool in QIIME2 (Version QIIME2-202006) to obtain initial ASVs, discarding those with an abundance under five. Species annotation was executed via the QIIME2 software, using the Silva Database for 16S/18S and the Unite Database for ITS sequences. QIIME2 also facilitated multiple sequence alignments to examine phylogenetic relations and dominant species variations across different samples or groups. Normalization of ASV abundances was based on the least sequenced sample, and both alpha and beta diversity analyses proceeded from this normalized data.

### Statistics

We performed statistical evaluations using SPSS version 26 (IBM, Armonk, New York, USA) along with RStudio (version 4.2.3, RStudio Inc., Boston, MA, USA). Within SPSS, we applied univariate non-parametric evaluations—specifically, Wilcoxon signed-rank, sigh, and marginal homogeneity tests—to discern disparities in exhaled VOC levels between individuals with CRC and healthy controls, considering a p-value below 0.05 as indicative of statistical significance. The relationship between respiratory and conventional serum biomarkers was explored through Spearman's correlation. Meanwhile, RStudio facilitated the use of linear discriminant analysis (LDA) to compress and cluster the VOC dataset.

## Supplementary Material

Supplementary figures and tables.

Supplementary data.
