# Supplementary material for: Pioneering noninvasive colorectal cancer detection with an AI-enhanced breath volatilomics platform
Source: Theranostics. 2024 Jul 8;14(11):4240–55. doi: 10.7150/thno.94950 (PMC11303087; doi:10.7150/thno.94950)
Supplement: Supplementary file 1 — Supplementary figures and tables. [file thnov14p4240s1.pdf]

# Supplementary material

## Pioneering noninvasive colorectal cancer detection with an AI-enhanced breath volatilomics platform

Yongqian Liu [1]†, Yongyan Ji[1]†, Jian Chen[1], Yixuan Zhang[2], Xiaowen Li[2 *], Xiang Li[1 *]

[1]Department of Environmental Science & Engineering, Fudan University, Shanghai 200438, P.R. China.

[2]Department of gastroenterology, Huadong hospital, Fudan University, Shanghai 200040, P.R. China.

*Corresponding author. Email: lixiang@fudan.edu.cn (XL)

†These authors contributed equally to this work.

**Supplementary Method:**

**Method S1.** Improvements to the breath sampler.

**Supplementary Tables:**

**Table S1.** Detailed patient demographics of colorectal cancer and control groups.

**Table S2.** PLS-DA VIP score.

**Table S3.** Presentation of the results of ANOVA analysis.

**Table S4.** Binary logistic analysis results.

**Table S5.** The 64 candidate VOCs list for subsequent model construction.

**Table S6.** Count results based on three feature selection algorithms.

**Table S7.** Feature importance list for Diagnostic Model.
**Table S8.** Baseline classifiers performance evaluation for Diagnostic Model.
**Table S9.** Feature importance list for Metastatic Model.
**Table S10.** Baseline classifiers performance evaluation for Metastatic Model.
**Table S11.** Prediction performance in Diagnostic Model.
**Table S12.** Prediction performance in Metastatic Model.

**Supplementary Figures:**

**Figure S1.** PCA analysis of VOCs between polyp and completely healthy groups.
**Figure S2.** Clustered heatmap of VOCs concentration between polyp and completely health groups.
**Figure S3.** Volcano diagrams of different groupings of substances in human exhalation.
**Figure S4.** Heatmaps of VOCs correlations in CRC.
**Figure S5.** Categorical relevance calculations by compounds groups.
**Figure S6.** The performance of Diagnostic Model in discerning LNM and DM from NM group.
**Figure S7.** The performance of traditional cancer serum markers.
**Figure S8.** The diagnosis efficiency of each marker among the fifteen markers in the diagnostic model.
**Figure S9.** Principal Co-ordinates Analysis Plots.
**Figure S10.** Seventeen statistically tested significantly different KEGG pathways.
**Figure S11.** The number of feature selection was determined by AUC and accuracy.

**Figure S12.** Diagram of participant recruitment and allocation ratios.

## Method S1. Improvements to the breath sampler

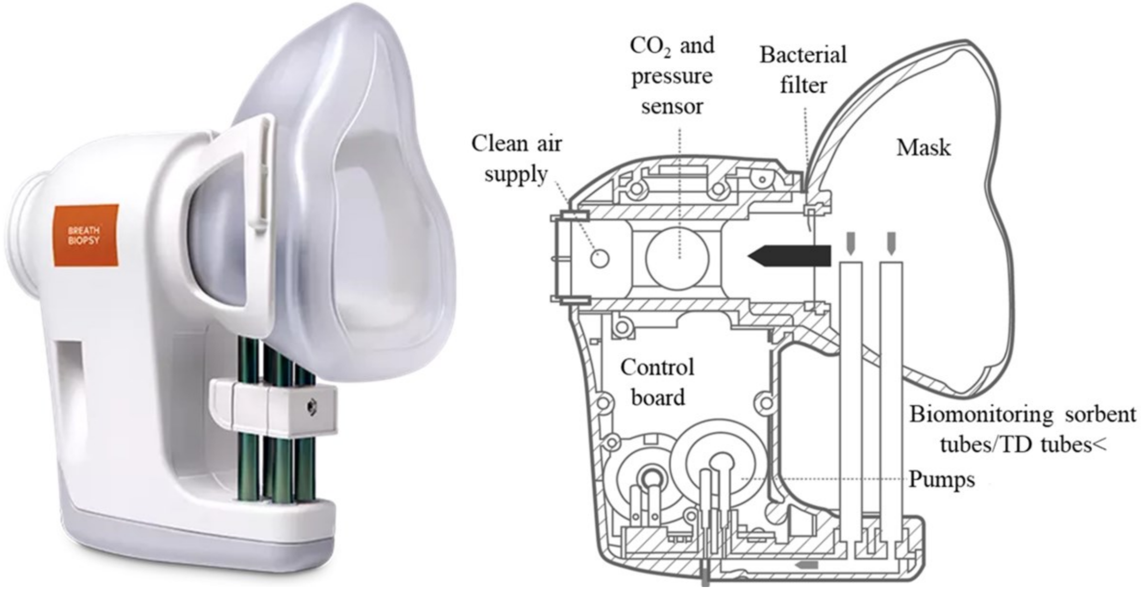


Method Figure S1. Schematic of the ReCIVA device. ®

### a) Optimization of sampler parameters

For the optimization of the respiratory sampler parameters, this study utilized the built-in temperature, pressure, and $CO_2$ sensors of the ReCIVA respiratory sampler from Owlstone Medical, U.K. The accompanying software learns the breathing pattern of the subject based on the online data from the three sensors for 20 seconds prior to sampling. It can be selected to collect the exhaled components of the specific phase, including the lower respiratory tract, upper respiratory tract, and the whole respiratory tract components. This study collected six parallel samples of various respiratory components from the same subject. The optimal components were selected based on instrumental analysis results. The sampling flow rate parameters were further optimized by collecting six breath samples from the same subject at different collection flow rates. Two adsorbent tubes were connected, and the internal standard was injected into the upper adsorbent tube. Different volumes of air were then pumped in using a hand-held pump to plot the penetration curve of the internal standard in the lower adsorbent tube. This allowed for the selection of an appropriate sampling volume to improve the method's detection limit. The VOCs were concentrated in adsorbent tubes (Tenax TA+Carbograph-5TD) using a modified breath sampler. A standardized sampling method was developed by quantifying interfering factors to ensure

reproducible results.

**b) Details of Sampler Construction**

The breath sampler's specific construction includes an air supply pump that filters background contaminated VOCs from ambient gases and provides pure air for the sampling process. Additionally, it features a sampling mask that fits snugly over the face and is connected to the breath sampler. The face mask is equipped with four holes, two on the left and two on the right side of the lower edge, where adsorption tubes can be inserted to collect VOCs from exhaled breath. After inserting the tubes, the mask should be fastened to the sampler interface. Please refer to Supplementary Method Figure 1 for more details.

**Table S1. Detailed patient demographics of colorectal cancer (CRC) and control groups.**

| Parameter | | CRC patients only number, (%) (n=101) | All Controls (non-CRC), number, (%) (n=93) | p value between CRC and control patients |
|---|---|---|---|---|
| No. of patients who had breath sampled | | 194 | | |
| No of patients excluded due to inadequate reference test (incomplete/cancelled colonoscopy) | | 2 | | |
| No. of patients excluded due to failure of QC for VOC presence in TD tubes | | 2 | | |
| No. of patients excluded due to Recovered from surgery or with alternate pathological diagnoses such as mucinous adenocarcinoma, and other non- CRC tumors | | 8 | | |
| Gender | Female | 40(44.4%) | 39(42.5%) | 0.134 |
| | Male | 50(55.6%) | 53(57.5%) | |
| | Unrecorded | 0 | 0 | |
| Age (years) | Median (IQR) | 67(13.75) | 61(15) | 0.051 |
| | Minimum - Maximum | 38-88 | 22-83 | |
| | Unrecorded | 1 | 0 | |
| BMI (kg/m$^2$) | Median (IQR) | 23.64(4) | 23.03(3.33) | 0.860 |
| | Minimum - Maximum | 18.2-30.8 | 17.6-51.4 | |

| | | | | |
|---|---|---|---|---|
| | Unrecorded | 8 | 3 | |
| Smoking Status | Current | 25(25.6%) | 22(23.9%) | 0.586 |
| | Ex | 3(3.33%) | 5(5.4%) | |
| | Never | 62(68.9%) | 65(70.7%) | |
| | Unrecorded | 0 | 2 | |
| Alcohol intake status | Current | 13(14.4%) | 12(13.0%) | 0.204 |
| | Ex | 2(2.22%) | 0 | |
| | Never | 74(82.2%) | 77(87.5%) | |
| | Unrecorded | 1 | 3 | |
| Past medical history | HBP* | 23(25.6%) | 15(16.3%) | 0.332 |
| | Chronic Obstructive Pulmonary Disease (COPD) | 3(3.3%) | 1(1.1%) | 0.426 |
| | Diabetes* | 16(17.8%) | 19(20.7%) | 0.861 |
| | Asthma | 0 | 1(1.1%) | 0.553 |
| | HBV | 4(4.4%) | 0 | 0.046 |
| | Renal impairment | 0 | 1(1.1%) | 0.920 |
| | Liver impairment | 3(3.3%) | 2(2.2%) | 0.801 |
| | Known heart disease | 1(1.1%) | 2(2.2%) | 0.635 |
| | Previous cancer excluding CRC* | 2(2.2%) | 3(3.3%) | 0.852 |
| Medications | CCB | 9(10.0%) | 5(5.4%) | 0.789 |
| | ARB | 7(7.8%) | 7(7.6%) | 0.006 |

| | | | | |
|---|---|---|---|---|
| | Sitagliptin | 1(1.1%) | 0 | 0.265 |
| | Bisoprolol | 1(1.1%) | 1(1.1%) | 0.471 |
| | Metformin | 2(2.2%) | 0 | 0.336 |
| | Glimepiride | 1(1.1%) | 0 | 0.737 |
| | Saxagliptin | 1(1.1%) | 0 | 0.296 |
| Patient-reported Symptoms | Rectal bleeding | 11(12.2%) | 0 | 0.240 |
| | Abdominal pain | 11(12.2%) | 6(6.5%) | 0.889 |
| | Bloating | 9(10.0%) | 3(3.3%) | 0.570 |
| | Weight loss | 6(6.7%) | 0 | 0.031 |
| | Appetite loss | 2(2.2%) | 0 | 0.651 |
| | Anemic | 3(3.3%) | 0 | 0.432 |
| | Dark stool | 5(5.6%) | 0 | 0.063 |
| | Bowel obstruction | 1(1.1%) | 0 | 0.940 |
| Tumor site | Left sided: rectum to splenic flexure | 56(62.7%) | NA | NA |
| | Right sided: transverse colon to caecum | 14(15.7%) | NA | NA |
| | Left sided and right sided | 20(22.2%) | NA | NA |
| Clinical tumor stage | I | 16(17.8%) | NA | NA |
| | II | 30(33.3%) | NA | NA |
| | III | 25(27.8%) | NA | NA |
| | IV | 19(21.1%) | NA | NA |

| | | | | |
|---|---|---|---|---|
| Clinical metastasis stage | 0 | 46(51.1%) | NA | NA |
| | 1 | 44(48.9%) | NA | NA |
| Differentiation of tumor | Well differentiated | 0 | NA | NA |
| | Moderately differentiated | 42(47.1%) | NA | NA |
| | Moderately to Poorly differentiated | 20(21.8%) | NA | NA |
| | Poorly differentiated | 28(25.1%) | NA | NA |

**Table S2. PLS-DA VIP score**

| VIP | Compound name |
|---|---|
| 1.91 | Methacrolein |
| 1.79 | 3-Furaldehyde |
| 1.79 | Isoprene |
| 1.56 | Thiophene-3-Methyl |
| 1.49 | o-Xylene |
| 1.47 | p-Xylene |
| 1.46 | Ethylbenzene |
| 1.40 | 2-Pentanone |
| 1.40 | Hexanal |
| 1.32 | 3-Thiophenecarboxaldehyde |
| 1.29 | 1-Butanol |
| 1.28 | Propanoic Acid |
| 1.27 | α-Methylstyrene |
| 1.26 | Cumene |
| 1.26 | 2-Methylfuran |
| 1.26 | Isobutyric Acid |
| 1.25 | Methyl Thiocyanate |
| 1.25 | Heptanal |
| 1.23 | Octanal |
| 1.20 | 1-Propene,1-Methylthio- |
| 1.20 | Dimethyl Disulfide |
| 1.14 | Acetone |
| 1.13 | Geranyl Acetone |
| 1.13 | Nonanal |
| 1.10 | Propanal |
| 1.09 | 1,4-Dioxane |
| 1.09 | Nonane |
| 1.09 | α-Pinene |
| 1.06 | Toluene |
| 1.04 | Sulfide,Allyl Methyl |
| 1.02 | 2-Heptanone |
| 1.00 | Octane,2-Methyl- |
| 1.00 | Camphene |

**Table S3. Presentation of the results of ANOVA analysis.**

| VOCs | Smoking status(avg±SD) | | F | p |
|---|---|---|---|---|
| | 0.0(n=57) | 1.0(n=14) | | |
| 2-Methylbutane | 8.18±7.24 | 11.21±7.18 | 1.976 | 0.044* |
| Isoprene | 382.72±228.06 | 421.61±207.82 | 0.338 | 0.563 |
| Acetone | 1670.13±1717.02 | 2121.01±1834.68 | 0.755 | 0.388 |
| Cyclopentane | 0.68±0.40 | 0.75±0.43 | 0.35 | 0.556 |
| Pentane, 2,4-Dimethyl- | 0.44±0.32 | 0.47±0.32 | 0.089 | 0.766 |
| Ethyl Acetate | 1.63±1.20 | 1.72±0.88 | 0.07 | 0.793 |
| Hexane, 2-Methyl- | 0.34±0.21 | 0.37±0.23 | 0.234 | 0.63 |
| 1-Butanol | 3.55±3.10 | 4.00±2.97 | 0.24 | 0.626 |
| 2-Pentanone | 1.94±1.34 | 2.19±1.34 | 0.372 | 0.544 |
| 1,4-Dioxane | 1.14±1.02 | 1.24±1.00 | 0.129 | 0.72 |
| Heptane, 3-Methyl- | 0.27±0.12 | 0.27±0.12 | 0.008 | 0.93 |
| Acetoin | 1.62±1.27 | 1.92±1.49 | 0.596 | 0.443 |
| Pyridine | 0.43±0.16 | 0.49±0.17 | 1.82 | 0.021* |
| 1,2-Propanediol | 2.89±2.19 | 2.59±1.68 | 0.23 | 0.633 |
| Cyclopentanone | 0.10±0.06 | 0.12±0.07 | 1.578 | 0.213 |
| Octane, 2-Methyl- | 0.30±0.16 | 0.29±0.16 | 0.008 | 0.928 |
| 2-Heptanone | 0.12±0.05 | 0.14±0.05 | 1 | 0.321 |
| 6-Methyl-5-Heptene-2-One | 1.42±0.89 | 2.13±1.20 | 6.196 | 0.015* |
| γ-Terpinene | 0.44±0.06 | 0.47±0.03 | 3.072 | 0.013* |
| 2-Nonanone | 0.06±0.04 | 0.06±0.04 | 0.089 | 0.767 |
| Geranyl Acetone | 2.87±2.32 | 3.74±2.95 | 1.401 | 0.241 |
| n-Pentane | 22.79±22.31 | 37.76±27.65 | 4.599 | 0.036* |
| Propanal | 1.66±1.35 | 3.06±1.49 | 11.733 | 0.041* |
| Hexane | 4.28±4.17 | 6.01±5.81 | 1.636 | 0.205 |
| Methacrolein | 6.03±2.51 | 6.03±2.23 | 0 | 0.996 |
| Heptane | 0.73±0.38 | 0.86±0.35 | 1.367 | 0.246 |
| Propanoic Acid | 14.82±12.92 | 28.10±15.98 | 10.806 | 0.141 |
| Octane | 0.42±0.16 | 0.39±0.16 | 0.489 | 0.487 |
| Isobutyric Acid | 2.19±1.77 | 3.29±1.77 | 4.298 | 0.042* |
| Hexanal | 1.00±0.65 | 0.99±0.52 | 0.004 | 0.952 |
| Butyric Acid | 2.50±1.96 | 5.08±2.44 | 17.675 | 0.034* |
| Nonane | 0.35±0.20 | 0.42±0.16 | 1.215 | 0.274 |
| Heptanal | 0.60±0.21 | 0.65±0.15 | 0.66 | 0.419 |
| Valeric Acid | 1.21±0.49 | 1.22±0.44 | 0.002 | 0.967 |
| Octanal | 0.62±0.24 | 0.76±0.24 | 4.226 | 0.044* |

continued

| | | | | |
|---|---|---|---|---|
| Hexanoic Acid | 1.23±0.49 | 1.39±0.57 | 1.05 | 0.309 |
| Nonanal | 1.68±0.74 | 1.96±0.69 | 1.64 | 0.205 |
| Decanal | 0.92±0.48 | 1.17±0.51 | 2.808 | 0.038* |
| Cyclohexane | 0.53±0.31 | 0.57±0.28 | 0.23 | 0.633 |
| 1-Propene, 1-(Methylthio)- | 0.18±0.17 | 0.27±0.24 | 2.636 | 0.009* |
| α-Pinene | 0.72±0.52 | 0.88±0.63 | 0.966 | 0.329 |
| α-Methylstyrene | 0.06±0.02 | 0.06±0.01 | 0.296 | 0.588 |
| Decane | 0.77±0.65 | 0.48±0.51 | 2.314 | 0.031* |
| Undecane | 0.49±0.41 | 0.79±0.63 | 4.838 | 0.033* |
| Dodecane | 0.95±0.70 | 1.00±0.92 | 0.062 | 0.804 |
| Tridecane | 0.60±0.43 | 0.55±0.46 | 0.149 | 0.7 |
| n-Tetradecane | 1.35±1.08 | 1.15±1.08 | 0.408 | 0.525 |
| Pentadecane | 1.46±1.25 | 1.40±1.24 | 0.03 | 0.863 |
| Camphene | 0.35±0.21 | 0.37±0.18 | 0.242 | 0.624 |
| 2-Methylfuran | 0.97±0.58 | 1.43±0.64 | 6.654 | 0.012* |
| Benzene | 4.17±1.37 | 5.01±1.63 | 3.984 | 0.050* |
| Sulfide, Allyl Methyl | 0.31±0.42 | 0.46±0.50 | 1.298 | 0.259 |
| Dimethyl Disulfide | 0.88±0.51 | 0.80±0.40 | 0.361 | 0.55 |
| Methyl Thiocyanate | 1.86±1.57 | 1.75±2.04 | 0.049 | 0.826 |
| Toluene | 3.68±2.60 | 5.21±2.18 | 4.128 | 0.046* |
| Thiophene, 3-Methyl- | 6.18±4.40 | 4.36±4.20 | 1.972 | 0.045* |
| 3-Furaldehyde | 2.31±1.17 | 2.08±0.69 | 0.532 | 0.468 |
| Ethylbenzene | 0.92±0.50 | 1.12±0.33 | 2.215 | 0.002** |
| p-Xylene | 0.93±0.51 | 1.14±0.42 | 1.984 | 0.037* |
| Furfural | 0.68±0.25 | 0.85±0.32 | 4.536 | 0.013* |
| o-Xylene | 0.79±0.41 | 0.91±0.29 | 1.16 | 0.035* |
| Cumene | 0.38±0.18 | 0.49±0.15 | 4.645 | 0.025* |
| Dimethyl Trisulfide | 0.80±0.27 | 0.85±0.35 | 0.318 | 0.575 |
| Benzaldehyde | 3.78±1.95 | 3.11±0.90 | 1.568 | 0.215 |
| Benzonitrile | 0.65±0.27 | 0.61±0.35 | 0.196 | 0.659 |
| 3-Thiophenecarboxaldehyde | 1.34±0.87 | 1.06±0.75 | 1.228 | 0.272 |
| 2-Thiophenecarboxaldehyde | 0.33±0.13 | 0.36±0.12 | 0.317 | 0.575 |
| Phenol | 2.91±1.64 | 2.58±1.26 | 0.507 | 0.479 |
| Acetophenone | 2.27±1.10 | 1.85±0.78 | 1.742 | 0.001** |
| m-Cresol | 0.29±0.20 | 0.34±0.23 | 0.584 | 0.448 |
| 4-Ethylphenol | 0.06±0.03 | 0.07±0.03 | 0.23 | 0.633 |
| D-Limonene | 2.41±1.89 | 2.44±1.42 | 0.005 | 0.945 |

* p<0.05 ** p<0.01

**Table S4. Binary logistic analysis results.** implicit variable: smoking status.

| Variables | regression coefficient | SD | z value | Wald χ2 | p | OR |
|---|---|---|---|---|---|---|
| 2-Methylbutane | -0.273 | 0.193 | -1.419 | 2.015 | 0.156 | 0.761 |
| Pyridine | 1.460 | 4.473 | 0.326 | 0.107 | 0.744 | 4.306 |
| 6-Methyl-5-Heptene-2-One | 2.312 | 1.306 | 1.771 | 3.135 | 0.747 | 10.093 |
| γ-Terpinene | 34.253 | 25.906 | 1.322 | 1.748 | 0.186 | 7.51159E+14 |
| n-Pentane | -0.050 | 0.058 | -0.856 | 0.733 | 0.392 | 0.951 |
| Propanal | 2.005 | 0.901 | 2.225 | 4.950 | 0.026 | 7.423 |
| Octanal | -6.427 | 7.119 | -0.903 | 0.815 | 0.367 | 0.002 |
| Decanal | 0.239 | 2.796 | 0.085 | 0.007 | 0.932 | 1.270 |
| 1-Propene, 1-(Methylthio)- | 7.687 | 4.903 | 1.568 | 2.458 | 0.117 | 2179.104 |
| Decane | -4.849 | 2.173 | -2.232 | 4.981 | 0.026 | 0.008 |
| Undecane | -0.559 | 2.46 | -0.227 | 0.052 | 0.82 | 0.572 |
| 2-Methylfuran | 2.546 | 1.674 | 1.521 | 2.312 | 0.018 | 12.751 |
| Benzene | 1.117 | 1.061 | 1.053 | 1.109 | 0.002 | 3.055 |
| Toluene | -1.341 | 1.016 | -1.320 | 1.742 | 0.008 | 0.262 |
| Thiophene, 3-Methyl- | 0.059 | 0.235 | 0.250 | 0.062 | 0.803 | 1.060 |
| Ethylbenzene | 8.453 | 8.406 | 1.006 | 1.011 | 0.015 | 4689.343 |
| p-Xylene | 2.002 | 5.645 | 0.355 | 0.126 | 0.023 | 7.401 |
| Furfural | 0.546 | 3.339 | 0.163 | 0.027 | 0.870 | 1.726 |
| o-Xylene | 1.105 | 6.562 | 0.168 | 0.028 | 0.006 | 3.019 |
| Cumene | -2.479 | 5.386 | -0.460 | 0.212 | 0.045 | 0.084 |
| Acetophenone | -2.058 | 1.187 | -1.734 | 3.007 | 0.083 | 0.128 |
| intercept | -25.635 | 13.996 | -1.832 | 3.355 | 0.067 | 0 |

McFadden $R^2$: 0.609

Cox & Snell $R^2$：0.454

Nagelkerke $R^2$：0.720

**Table S5. The 64 candidate VOCs list for subsequent model construction.**

| No. | 64 Candidate VOCs | Chemical Classes | Abbreviation |
|---|---|---|---|
| 1 | 2-Methylbutane | Hydrocarbons | ISOP |
| 2 | isoprene | Hydrocarbons | IP |
| 3 | Acetone | Ketones | ACE |
| 4 | Cyclopentane | Hydrocarbons | CP |
| 5 | Pentane, 2,4-dimethyl- | Hydrocarbons | 24DP |
| 6 | Ethyl Acetate | Other oxygen-containing VOCs | EtAc |
| 7 | Hexane, 2-methyl- | Hydrocarbons | 2MHEX |
| 8 | 1-Butanol | Alcohols | 1BL |
| 9 | 2-pentanone | Ketones | 2PN |
| 10 | 1,4-Dioxane | Other oxygen-containing VOCs | Diox |
| 11 | Heptane, 3-methyl- | Hydrocarbons | 3MHP |
| 12 | acetoin | Alcohols | ACT |
| 13 | pyridine | Nitrogen-containing VOCs | Pyr |
| 14 | 1,2-Propanediol | Alcohols | PGO |
| 15 | Cyclopentanone | Ketones | CPO |
| 16 | Octane, 2-methyl- | Hydrocarbons | 2MOct |
| 17 | 2-Heptanone | Ketones | 2Hp |
| 18 | 6-Methyl-5-heptene-2-one | Ketones | 0VT |
| 19 | γ-Terpinene | Terpenes / Terpenoids | Gtp |
| 20 | 2-Nonanone | Ketones | 2Nn |
| 21 | geranyl acetone | Ketones | GACE |
| 22 | n-Pentane | Hydrocarbons | Pen |
| 23 | Propanal | Aldehydes | PA |
| 24 | Hexane | Hydrocarbons | Hex |
| 25 | Methacroleine | Aldehyde | MAC |
| 26 | Heptane | Hydrocarbons | Hep |
| 27 | propanoic acid | Fatty acids | PPA |
| 28 | Octane | Hydrocarbons | Oct |
| 29 | Isobutyric acid | Fatty acids | IBA |
| 30 | Hexanal | Aldehydes | HEX |
| 31 | Butyric acid | Fatty acids | BuA |
| 32 | Nonane | Hydrocarbons | N9 |
| 33 | Heptanal | Aldehydes | HEP |
| 34 | Valeric Acid | Fatty acids | Vla |

Continued

| | | | |
|---|---|---|---|
| 35 | Octanal | Aldehydes | OC |
| 36 | Hexanoic acid | Fatty acids | HEXA |
| 37 | Nonanal | Aldehydes | Non |
| 38 | Decanal | Aldehydes | Dec |
| 39 | Cyclohexane | Hydrocarbons | CHX |
| 40 | 1-Propene, 1-(methylthio)- | Sulphur-containing VOCs | 1M1P |
| 41 | α-Pinene | Terpenes / Terpenoids | 2PNE |
| 42 | α-Methylstyrene | Aromatics | aML |
| 43 | Undecane | Hydrocarbons | U11 |
| 44 | Dodecane | Hydrocarbons | D12 |
| 45 | tridecane | Hydrocarbons | T13 |
| 46 | n-tetradecane | Hydrocarbons | T14 |
| 47 | Pentadecane | Hydrocarbons | P15 |
| 48 | Camphene | Terpenes / Terpenoids | CPN |
| 49 | Sulfide, allyl methyl | Sulphur-containing VOCs | AMS |
| 50 | Dimethyl Disulfide | Sulphur-containing VOCs | DMDS |
| 51 | Methyl thiocyanate | Sulphur-containing VOCs | MTCN |
| 52 | Thiophene, 3-methyl- | Sulphur-containing VOCs | 3MTh |
| 53 | 3-Furaldehyde | Aldehydes | 3FD |
| 54 | Furfural | Aldehydes | FFu |
| 55 | Cumene | Aromatics | IPB |
| 56 | Dimethyl trisulfide | Sulphur-containing VOCs | DMTS |
| 57 | Benzaldehyde | Aldehydes | BAL |
| 58 | Benzonitrile | Nitrogen-containing VOCs | BN |
| 59 | 3-Thiophenecarboxaldehyde | Aldehydes | 3TPD |
| 60 | 2-Thiophenecarboxaldehyde | Aldehydes | 2TPD |
| 61 | Phenol | Phenols | Phe |
| 62 | m-Cresol | Phenols | MC |
| 63 | 4-Ethylphenol | Phenols | 4EP |
| 64 | DL-Limonene | Terpenes / Terpenoids | DL |

**Table S6. Count results based on three feature selection algorithms.**

| No. | features | boruta | LASSO | SVM-RFE |
|---|---|---|---|---|
| 1 | 2-Methylbutane | 94 | 62 | 24 |
| 2 | Isoprene | 0 | 0 | 14 |
| 3 | Acetone | 100 | 17 | 12 |
| 4 | Cyclopentane | 0 | 0 | 35 |
| 5 | Pentane,2,4-Dimethyl. | 70 | 0 | 24 |
| 6 | Ethyl Acetate | 93 | 0 | 44 |
| 7 | Hexane, 2-Methyl. | 100 | 0 | 55 |
| 8 | 1-Butanol | 0 | 46 | 29 |
| 9 | 2-Pentanone | 5 | 100 | 81 |
| 10 | 1,4-Dioxane | 100 | 93 | 83 |
| 11 | Heptane,3-Methyl. | 0 | 0 | 23 |
| 12 | Acetoin | 0 | 93 | 60 |
| 13 | Pyridine | 0 | 0 | 46 |
| 14 | 1,2-Propanediol | 0 | 11 | 15 |
| 15 | Cyclopentanone | 0 | 25 | 31 |
| 16 | Octane, 2-Methyl. | 100 | 100 | 98 |
| 17 | 2-Heptanone | 0 | 0 | 25 |
| 18 | 6-Methyl-5-Heptene-2-One | 52 | 0 | 14 |
| 19 | γ-Terpinene | 99 | 0 | 72 |
| 20 | 2-Nonanone | 100 | 86 | 47 |
| 21 | Geranyl Acetone | 100 | 100 | 96 |
| 22 | n-Pentane | 0 | 0 | 20 |
| 23 | Propanal | 0 | 0 | 16 |
| 24 | Hexane | 0 | 72 | 39 |
| 25 | Methacrolein | 0 | 0 | 13 |
| 26 | Heptane | 0 | 0 | 19 |
| 27 | Propanoic Acid | 0 | 0 | 53 |
| 28 | Octane | 0 | 0 | 13 |
| 29 | Isobutyric Acid | 0 | 0 | 63 |
| 30 | Hexanal | 0 | 25 | 9 |
| 31 | Butyric Acid | 95 | 100 | 64 |
| 32 | Nonane | 0 | 0 | 46 |
| 33 | Heptanal | 0 | 0 | 27 |

| | | | | continued |
|---|---|---|---|---|
| 34 | Valeric Acid | 100 | 100 | 99 |
| 35 | Octanal | 0 | 0 | 12 |
| 36 | Hexanoic Acid | 87 | 86 | 77 |
| 37 | Nonanal | 0 | 0 | 24 |
| 38 | Decanal | 0 | 0 | 14 |
| 39 | Cyclohexane | 100 | 0 | 60 |
| 40 | 1-Propene,1-Methylthio- | 0 | 0 | 36 |
| 41 | α-Pinene | 0 | 0 | 33 |
| 42 | α-Methylstyrene | 0 | 0 | 13 |
| 43 | Undecane | 0 | 0 | 22 |
| 44 | Dodecane | 100 | 0 | 98 |
| 45 | Tridecane | 0 | 0 | 78 |
| 46 | n-Tetradecane | 100 | 60 | 97 |
| 47 | Pentadecane | 100 | 0 | 99 |
| 48 | Camphene | 0 | 0 | 28 |
| 49 | Sulfide, Allyl Methyl | 100 | 100 | 91 |
| 50 | Dimethyl Disulfide | 89 | 9 | 26 |
| 51 | Methyl Thiocyanate | 0 | 4 | 52 |
| 52 | Thiophene, 3-Methyl- | 89 | 100 | 96 |
| 53 | 3-Furaldehyde | 0 | 17 | 42 |
| 54 | Furfural | 100 | 100 | 100 |
| 55 | Dimethyl Trisulfide | 100 | 0 | 46 |
| 56 | Benzaldehyde | 100 | 100 | 99 |
| 57 | Benzonitrile | 76 | 100 | 80 |
| 58 | 3-Thiophenecarboxaldehyde | 0 | 0 | 34 |
| 59 | 2-Thiophenecarboxaldehyde | 91 | 0 | 94 |
| 60 | Phenol | 0 | 0 | 47 |
| 61 | Acetophenone | 95 | 40 | 94 |
| 62 | m-Cresol | 0 | 0 | 30 |
| 63 | 4-Ethylphenol | 0 | 0 | 32 |
| 64 | D-Limonene | 100 | 93 | 31 |

**Table S7. Feature importance list for Diagnostic Model.**

| Order | No. | features | sum |
|---|---|---|---|
| **1** | 54 | **Furfural** | 300 |
| **2** | 34 | **Valeric Acid** | 299 |
| **3** | 56 | **Benzaldehyde** | 299 |
| **4** | 16 | **Octane, 2-Methyl-** | 298 |
| **5** | 21 | **Geranyl Acetone** | 296 |
| **6** | 49 | **Sulfide, Allyl Methyl** | 291 |
| **7** | 52 | **Thiophene, 3-Methyl-** | 285 |
| **8** | 10 | **1,4-Dioxane** | 276 |
| **9** | 31 | **Butyric Acid** | 259 |
| **10** | 46 | **n-Tetradecane** | 257 |
| **11** | 57 | **Benzonitrile** | 256 |
| **12** | 36 | **Hexanoic Acid** | 250 |
| **13** | 20 | **2-Nonanone** | 233 |
| **14** | 64 | **D-Limonene** | 224 |
| 15 | 47 | Pentadecane | 199 |
| 16 | 44 | Dodecane | 198 |
| 17 | 9 | 2-Pentanone | 186 |
| 18 | 59 | 2-Thiophenecarboxaldehyde | 185 |
| 19 | 1 | 2-Methylbutane | 180 |
| 20 | 19 | γ-Terpinene | 171 |
| 21 | 39 | Cyclohexane | 160 |
| 22 | 7 | Hexane, 2-Methyl- | 155 |
| 23 | 12 | Acetoin | 153 |
| 24 | 61 | Acetophenone | 150 |
| 25 | 55 | Dimethyl Trisulfide | 146 |
| 26 | 6 | Ethyl Acetate | 137 |
| 27 | 3 | Acetone | 129 |
| 28 | 50 | Dimethyl Disulfide | 124 |
| 29 | 24 | Hexane | 111 |
| 30 | 5 | Pentane,2,4-Dimethyl- | 94 |
| 31 | 45 | Tridecane | 78 |
| 32 | 8 | 1-Butanol | 75 |

| | | | continued |
|---|---|---|---|
| 33 | 18 | 6-Methyl-5-Heptene-2-One | 66 |
| 34 | 29 | Isobutyric Acid | 63 |
| 35 | 53 | 3-Furaldehyde | 59 |
| 36 | 15 | Cyclopentanone | 56 |
| 37 | 51 | Methyl Thiocyanate | 56 |
| 38 | 27 | Propanoic Acid | 53 |
| 39 | 60 | Phenol | 47 |
| 40 | 13 | Pyridine | 46 |
| 41 | 32 | Nonane | 46 |
| 42 | 40 | 1-Propene,1-Methylthio- | 36 |
| 43 | 4 | Cyclopentane | 35 |
| 44 | 30 | Hexanal | 34 |
| 45 | 58 | 3-Thiophenecarboxaldehyde | 34 |
| 46 | 41 | α-Pinene | 33 |
| 47 | 63 | 4-Ethylphenol | 32 |
| 48 | 62 | m-Cresol | 30 |
| 49 | 48 | Camphene | 28 |
| 50 | 33 | Heptanal | 27 |
| 51 | 14 | 1,2-Propanediol | 26 |
| 52 | 17 | 2-Heptanone | 25 |
| 53 | 37 | Nonanal | 24 |
| 54 | 11 | Heptane,3-Methyl. | 23 |
| 55 | 43 | Undecane | 22 |
| 56 | 22 | n-Pentane | 20 |
| 57 | 26 | Heptane | 19 |
| 58 | 23 | Propanal | 16 |
| 59 | 2 | Isoprene | 14 |
| 60 | 38 | Decanal | 14 |
| 61 | 25 | Methacrolein | 13 |
| 62 | 28 | Octane | 13 |
| 63 | 42 | α-Methylstyrene | 13 |
| 64 | 35 | Octanal | 12 |

**Table S8. Baseline classifiers performance evaluation for Diagnostic Model.**

| NNet | | | LR | | | RF | | | SVM | | | XGB | | |
|---|---|---|---|---|---|---|---|---|---|---|---|---|---|---|
| **No.** | **Auc** | **Acc** | **No.** | **Auc** | **Acc** | **No.** | **Auc** | **Acc** | **No.** | **Auc** | **Acc** | **No.** | **Auc** | **Acc** |
| 1 | 0.567 | 0.618 | 1 | 0.729 | 0.778 | 1 | 0.694 | 0.667 | 1 | 0.494 | 0.519 | 1 | 0.448 | 0.480 |
| 2 | 0.633 | 0.676 | 2 | 0.813 | 0.833 | 2 | 0.544 | 0.556 | 2 | 0.603 | 0.630 | 2 | 0.628 | 0.680 |
| 3 | 0.674 | 0.706 | 3 | 0.771 | 0.806 | 3 | 0.594 | 0.593 | 3 | 0.603 | 0.630 | 3 | 0.573 | 0.640 |
| 4 | 0.767 | 0.794 | 4 | 0.750 | 0.778 | 4 | 0.624 | 0.630 | 4 | 0.703 | 0.704 | 4 | 0.604 | 0.680 |
| 5 | 0.761 | 0.765 | 5 | 0.771 | 0.806 | 5 | 0.653 | 0.667 | 5 | 0.653 | 0.667 | 5 | 0.684 | 0.720 |
| 6 | 0.774 | 0.794 | 6 | 0.813 | 0.833 | 6 | 0.682 | 0.704 | 6 | 0.653 | 0.667 | 6 | 0.549 | 0.640 |
| 7 | 0.847 | 0.853 | 7 | 0.708 | 0.750 | 7 | 0.712 | 0.741 | 7 | 0.732 | 0.741 | 7 | 0.660 | 0.720 |
| 8 | 0.847 | 0.853 | 8 | 0.792 | 0.806 | 8 | 0.712 | 0.741 | 8 | 0.732 | 0.741 | 8 | 0.771 | 0.800 |
| 9 | 0.874 | 0.882 | 9 | 0.833 | 0.833 | 9 | 0.712 | 0.741 | 9 | 0.732 | 0.741 | 9 | 0.604 | 0.680 |
| 10 | 0.814 | 0.824 | 10 | 0.833 | 0.833 | 10 | 0.712 | 0.741 | 10 | 0.732 | 0.741 | 10 | 0.604 | 0.680 |
| 11 | 0.874 | 0.882 | 11 | 0.875 | 0.861 | 11 | 0.712 | 0.741 | 11 | 0.732 | 0.741 | 11 | 0.660 | 0.720 |
| 12 | 0.847 | 0.853 | 12 | 0.771 | 0.750 | 12 | 0.712 | 0.741 | 12 | 0.732 | 0.741 | 12 | 0.722 | 0.800 |
| 13 | 0.881 | 0.882 | 13 | 0.792 | 0.778 | 13 | 0.712 | 0.741 | 13 | 0.821 | 0.852 | 13 | 0.722 | 0.800 |
| **14** | **0.907** | **0.912** | 14 | 0.792 | 0.778 | 14 | 0.712 | 0.741 | 14 | 0.791 | 0.815 | 14 | 0.722 | 0.800 |
| 15 | 0.874 | 0.882 | 15 | 0.792 | 0.806 | 15 | 0.762 | 0.778 | 15 | 0.791 | 0.815 | 15 | 0.691 | 0.760 |
| 16 | 0.847 | 0.853 | 16 | 0.771 | 0.778 | 16 | 0.762 | 0.778 | 16 | 0.741 | 0.778 | 16 | 0.691 | 0.760 |
| 17 | 0.833 | 0.853 | 17 | 0.813 | 0.833 | 17 | 0.732 | 0.741 | 17 | 0.741 | 0.778 | 17 | 0.635 | 0.720 |

continued

| | | | | | | | | | | | | | | |
|---|---|---|---|---|---|---|---|---|---|---|---|---|---|---|
| 18 | 0.833 | 0.853 | 18 | 0.771 | 0.750 | 18 | 0.682 | 0.704 | 18 | 0.712 | 0.741 | 18 | 0.604 | 0.680 |
| 19 | 0.781 | 0.794 | 19 | 0.771 | 0.750 | 19 | 0.653 | 0.667 | 19 | 0.712 | 0.741 | 19 | 0.611 | 0.720 |
| 20 | 0.781 | 0.794 | 20 | 0.750 | 0.750 | 20 | 0.732 | 0.741 | 20 | 0.791 | 0.815 | 20 | 0.611 | 0.720 |
| 21 | 0.867 | 0.882 | 21 | 0.833 | 0.833 | 21 | 0.682 | 0.704 | 21 | 0.791 | 0.815 | 21 | 0.611 | 0.720 |
| 22 | 0.714 | 0.735 | 22 | 0.833 | 0.833 | 22 | 0.732 | 0.741 | 22 | 0.791 | 0.815 | 22 | 0.611 | 0.720 |
| 23 | 0.747 | 0.765 | 23 | 0.729 | 0.750 | 23 | 0.682 | 0.704 | 23 | 0.791 | 0.815 | 23 | 0.722 | 0.800 |
| 24 | 0.840 | 0.853 | 24 | 0.688 | 0.667 | 24 | 0.712 | 0.741 | 24 | 0.791 | 0.815 | 24 | 0.722 | 0.800 |
| 25 | 0.688 | 0.706 | 25 | 0.792 | 0.806 | 25 | 0.732 | 0.741 | 25 | 0.741 | 0.778 | 25 | 0.722 | 0.800 |
| 26 | 0.847 | 0.853 | 26 | 0.729 | 0.750 | 26 | 0.732 | 0.741 | 26 | 0.771 | 0.815 | 26 | 0.722 | 0.800 |
| 27 | 0.781 | 0.794 | 27 | 0.688 | 0.694 | 27 | 0.741 | 0.778 | 27 | 0.771 | 0.815 | 27 | 0.667 | 0.760 |
| 28 | 0.747 | 0.765 | 28 | 0.771 | 0.750 | 28 | 0.791 | 0.815 | 28 | 0.821 | 0.852 | 28 | 0.858 | 0.880 |
| 29 | 0.747 | 0.765 | 29 | 0.833 | 0.833 | 29 | 0.712 | 0.741 | 29 | 0.771 | 0.815 | 29 | 0.771 | 0.800 |
| 30 | 0.781 | 0.794 | 30 | 0.729 | 0.750 | 30 | 0.741 | 0.778 | 30 | 0.771 | 0.815 | 30 | 0.635 | 0.720 |
| 31 | 0.767 | 0.794 | 31 | 0.729 | 0.750 | 31 | 0.741 | 0.778 | 31 | 0.741 | 0.778 | 31 | 0.635 | 0.720 |
| 32 | 0.714 | 0.735 | 32 | 0.708 | 0.750 | 32 | 0.741 | 0.778 | 32 | 0.821 | 0.852 | 32 | 0.691 | 0.760 |
| 33 | 0.728 | 0.735 | 33 | 0.604 | 0.667 | 33 | 0.712 | 0.741 | 33 | 0.821 | 0.852 | 33 | 0.691 | 0.760 |
| 34 | 0.728 | 0.735 | 34 | 0.688 | 0.722 | 34 | 0.741 | 0.778 | 34 | 0.821 | 0.852 | 34 | 0.747 | 0.800 |
| 35 | 0.728 | 0.735 | 35 | 0.750 | 0.778 | 35 | 0.791 | 0.815 | 35 | 0.791 | 0.815 | 35 | 0.778 | 0.840 |
| 36 | 0.774 | 0.794 | 36 | 0.583 | 0.583 | 36 | 0.741 | 0.778 | 36 | 0.721 | 0.778 | 36 | 0.660 | 0.720 |
| 37 | 0.754 | 0.765 | 37 | 0.667 | 0.722 | 37 | 0.741 | 0.778 | 37 | 0.771 | 0.815 | 37 | 0.660 | 0.720 |
| 38 | 0.821 | 0.824 | 38 | 0.646 | 0.694 | 38 | 0.741 | 0.778 | 38 | 0.721 | 0.778 | 38 | 0.660 | 0.720 |

continued

| | | | | | | | | | | | | | | |
|---|---|---|---|---|---|---|---|---|---|---|---|---|---|---|
| 39 | 0.688 | 0.706 | 39 | 0.625 | 0.639 | 39 | 0.741 | 0.778 | 39 | 0.771 | 0.815 | 39 | 0.660 | 0.720 |
| 40 | 0.747 | 0.765 | 40 | 0.583 | 0.611 | 40 | 0.741 | 0.778 | 40 | 0.771 | 0.815 | 40 | 0.747 | 0.800 |
| 41 | 0.721 | 0.735 | 41 | 0.729 | 0.750 | 41 | 0.741 | 0.778 | 41 | 0.771 | 0.815 | 41 | 0.747 | 0.800 |
| 42 | 0.747 | 0.765 | 42 | 0.583 | 0.583 | 42 | 0.741 | 0.778 | 42 | 0.771 | 0.815 | 42 | 0.660 | 0.720 |
| 43 | 0.788 | 0.794 | 43 | 0.604 | 0.611 | 43 | 0.741 | 0.778 | 43 | 0.771 | 0.815 | 43 | 0.660 | 0.720 |
| 44 | 0.814 | 0.824 | 44 | 0.688 | 0.694 | 44 | 0.741 | 0.778 | 44 | 0.821 | 0.852 | 44 | 0.715 | 0.760 |
| 45 | 0.781 | 0.794 | 45 | 0.688 | 0.694 | 45 | 0.741 | 0.778 | 45 | 0.821 | 0.852 | 45 | 0.747 | 0.800 |
| 46 | 0.761 | 0.765 | 46 | 0.708 | 0.722 | 46 | 0.741 | 0.778 | 46 | 0.721 | 0.778 | 46 | 0.747 | 0.800 |
| 47 | 0.628 | 0.647 | 47 | 0.604 | 0.583 | 47 | 0.741 | 0.778 | 47 | 0.721 | 0.778 | 47 | 0.747 | 0.800 |
| 48 | 0.735 | 0.735 | 48 | 0.625 | 0.611 | 48 | 0.741 | 0.778 | 48 | 0.721 | 0.778 | 48 | 0.740 | 0.760 |
| 49 | 0.761 | 0.765 | 49 | 0.625 | 0.611 | 49 | 0.741 | 0.778 | 49 | 0.762 | 0.778 | 49 | 0.740 | 0.760 |
| 50 | 0.840 | 0.853 | 50 | 0.688 | 0.694 | 50 | 0.712 | 0.741 | 50 | 0.741 | 0.778 | 50 | 0.740 | 0.760 |
| 51 | 0.754 | 0.765 | 51 | 0.729 | 0.722 | 51 | 0.682 | 0.704 | 51 | 0.741 | 0.778 | 51 | 0.740 | 0.760 |
| 52 | 0.795 | 0.794 | 52 | 0.646 | 0.639 | 52 | 0.741 | 0.778 | 52 | 0.741 | 0.778 | 52 | 0.740 | 0.760 |
| 53 | 0.754 | 0.765 | 53 | 0.625 | 0.611 | 53 | 0.741 | 0.778 | 53 | 0.741 | 0.778 | 53 | 0.740 | 0.760 |
| 54 | 0.668 | 0.676 | 54 | 0.625 | 0.611 | 54 | 0.712 | 0.741 | 54 | 0.741 | 0.778 | 54 | 0.740 | 0.760 |
| 55 | 0.788 | 0.794 | 55 | 0.667 | 0.639 | 55 | 0.741 | 0.778 | 55 | 0.691 | 0.741 | 55 | 0.740 | 0.760 |
| 56 | 0.654 | 0.676 | 56 | 0.667 | 0.639 | 56 | 0.741 | 0.778 | 56 | 0.691 | 0.741 | 56 | 0.740 | 0.760 |
| 57 | 0.781 | 0.794 | 57 | 0.688 | 0.667 | 57 | 0.741 | 0.778 | 57 | 0.691 | 0.741 | 57 | 0.715 | 0.760 |
| 58 | 0.500 | 0.794 | 58 | 0.667 | 0.639 | 58 | 0.741 | 0.778 | 58 | 0.691 | 0.741 | 58 | 0.715 | 0.760 |
| 59 | 0.735 | 0.735 | 59 | 0.604 | 0.611 | 59 | 0.741 | 0.778 | 59 | 0.662 | 0.704 | 59 | 0.715 | 0.760 |

continued

| | | | | | | | | | | | | | | |
|---|---|---|---|---|---|---|---|---|---|---|---|---|---|---|
| 60 | 0.488 | 0.529 | 60 | 0.604 | 0.583 | 60 | 0.741 | 0.778 | 60 | 0.662 | 0.704 | 60 | 0.715 | 0.760 |
| 61 | 0.416 | 0.441 | 61 | 0.604 | 0.583 | 61 | 0.741 | 0.778 | 61 | 0.662 | 0.704 | 61 | 0.715 | 0.760 |
| 62 | 0.721 | 0.735 | 62 | 0.604 | 0.583 | 62 | 0.712 | 0.741 | 62 | 0.662 | 0.704 | 62 | 0.715 | 0.760 |
| 63 | 0.474 | 0.529 | 63 | 0.604 | 0.583 | 63 | 0.741 | 0.778 | 63 | 0.691 | 0.741 | 63 | 0.715 | 0.760 |
| 64 | 0.474 | 0.529 | 64 | 0.604 | 0.611 | 64 | 0.712 | 0.741 | 64 | 0.662 | 0.704 | 64 | 0.660 | 0.720 |

**Table S9. Feature importance list for Metastasis Model.**

| Order | features | boruta | LASSO | SVM-RFE | sum |
|---|---|---|---|---|---|
| **1** | **Octane, 2-Methyl-** | 80 | 3 | 41 | 124 |
| **2** | **Benzaldehyde** | 2 | 58 | 61 | 121 |
| **3** | **Furfural** | 0 | 55 | 60 | 115 |
| **4** | **Hexanoic Acid** | 2 | 51 | 55 | 108 |
| **5** | **Geranyl Acetone** | 0 | 58 | 47 | 105 |
| **6** | **Sulfide, Allyl Methyl** | 1 | 58 | 28 | 87 |
| **7** | **2-Nonanone** | 1 | 43 | 24 | 68 |
| 8 | 1,4-Dioxane | 0 | 0 | 43 | 43 |
| 9 | Valeric Acid | 1 | 23 | 18 | 42 |
| 10 | Benzonitrile | 0 | 23 | 18 | 41 |
| 11 | Butyric Acid | 0 | 18 | 18 | 36 |
| 12 | D-Limonene | 0 | 3 | 30 | 33 |
| 13 | Thiophene, 3-Methyl- | 0 | 3 | 17 | 20 |
| 14 | n-Tetradecane | 0 | 0 | 18 | 18 |

**Table S10. Baseline classifiers performance evaluation for Metastatic Model.**

| NNet | | | LR | | | RF | | | SVM | | | XGB | | |
|---|---|---|---|---|---|---|---|---|---|---|---|---|---|---|
| **No.** | **Auc** | **Acc** | **No.** | **Auc** | **Acc** | **No.** | **Auc** | **Acc** | **No.** | **Auc** | **Acc** | **No.** | **Auc** | **Acc** |
| 1 | 0.500 | #N/A | 1 | 0.500 | #N/A | 1 | 0.514 | 0.529 | 1 | 0.514 | 0.529 | 1 | 0.607 | 0.615 |
| 2 | 0.409 | 0.529 | 2 | 0.506 | 0.632 | 2 | 0.514 | 0.529 | 2 | 0.451 | 0.471 | 2 | 0.452 | 0.462 |
| 3 | 0.409 | 0.529 | 3 | 0.641 | 0.632 | 3 | 0.514 | 0.529 | 3 | 0.563 | 0.588 | 3 | 0.536 | 0.538 |
| 4 | 0.492 | 0.588 | 4 | 0.641 | 0.632 | 4 | 0.514 | 0.529 | 4 | 0.625 | 0.647 | 4 | 0.619 | 0.615 |
| 5 | 0.614 | 0.647 | 5 | 0.635 | 0.684 | 5 | 0.403 | 0.412 | 5 | 0.500 | 0.529 | 5 | 0.619 | 0.615 |
| 6 | 0.697 | 0.706 | 6 | 0.590 | 0.684 | 6 | 0.576 | 0.588 | 6 | 0.451 | 0.471 | 6 | 0.536 | 0.538 |
| 7 | 0.742 | 0.765 | 7 | 0.468 | 0.579 | 7 | 0.632 | 0.647 | 7 | 0.389 | 0.412 | **7** | **0.857** | **0.846** |
| 8 | 0.742 | 0.765 | 8 | 0.468 | 0.579 | 8 | 0.625 | 0.647 | 8 | 0.389 | 0.412 | 8 | 0.774 | 0.769 |
| 9 | 0.742 | 0.765 | 9 | 0.468 | 0.579 | 9 | 0.632 | 0.647 | 9 | 0.389 | 0.412 | 9 | 0.619 | 0.615 |
| 10 | 0.742 | 0.765 | 10 | 0.385 | 0.526 | 10 | 0.694 | 0.706 | 10 | 0.389 | 0.412 | 10 | 0.607 | 0.385 |
| 11 | 0.447 | 0.529 | 11 | 0.346 | 0.474 | 11 | 0.521 | 0.529 | 11 | 0.389 | 0.412 | 11 | 0.548 | 0.538 |
| 12 | 0.568 | 0.588 | 12 | 0.346 | 0.474 | 12 | 0.451 | 0.471 | 12 | 0.444 | 0.471 | 12 | 0.548 | 0.538 |
| 13 | 0.523 | 0.529 | 13 | 0.391 | 0.474 | 13 | 0.563 | 0.588 | 13 | 0.444 | 0.471 | 13 | 0.548 | 0.538 |
| 14 | 0.492 | 0.588 | 14 | 0.391 | 0.474 | 14 | 0.444 | 0.471 | 14 | 0.444 | 0.471 | 14 | 0.548 | 0.538 |

**Table S11. Prediction performance in Diagnostic Model.**

| | Classifiers | Target | Sensitivity | Specificity | Accuracy | AUC | Num of Fs |
|---|---|---|---|---|---|---|---|
| **Diagnostic Model** | Logistic Regression | **CRC** | 0.74 | 0.82 | 0.86 | 0.88 | 11 |
| | Random Forest | | 0.71 | 0.83 | 0.82 | 0.79 | 28 |
| | Support Vector Machine | | 0.72 | 0.85 | 0.82 | 0.85 | 13 |
| | Extreme Gradient Boosting | | 0.72 | 0.85 | 0.88 | 0.86 | 28 |
| | **Neural Networks** | | **0.81** | **0.88** | **0.91** | **0.91** | **14** |

| Rank | Feature | Category | Comment |
|---|---|---|---|
| 1 | Octane, 2-Methyl- | VOCs | Hydrocarbons |
| 2 | Furfural | VOCs | Aldehydes |
| 3 | Benzaldehyde | VOCs | Aldehydes |
| 4 | Valeric Acid | VOCs | Fatty acids |
| 5 | Hexanoic Acid | VOCs | Fatty acids |
| 6 | Sulfide, Allyl Methyl | VOCs | Sulphur-containing VOCs |
| 7 | Geranyl Acetone | VOCs | Ketones |
| 8 | Benzonitrile | VOCs | Nitrogen-containing VOCs |
| 9 | Cumene | VOCs | Aromatics |
| 10 | 1,4-Dioxane | VOCs | Other oxygen-containing VOCs |
| 11 | Butyric Acid | VOCs | Fatty acids |
| 12 | n-Tetradecane | VOCs | Hydrocarbons |
| 13 | 2-Nonanone | VOCs | Ketones |
| 14 | Thiophene, 3-Methyl- | VOCs | Sulphur-containing VOCs |

**Table S12. Prediction performance in Metastatic Model.**

| | Classifiers | Target | Sensitivity | Specificity | Accuracy | AUC | Num of Fs |
|---|---|---|---|---|---|---|---|
| **Metastatic Model** | Logistic Regression | **Advanced Cancer** | 0.68 | 0.72 | 0.63 | 0.64 | 3 |
| | Random Forest | | 0.71 | 0.70 | 0.71 | 0.69 | 10 |
| | Support Vector Machine | | 0.61 | 0.64 | 0.65 | 0.63 | 4 |
| | **Extreme Gradient Boosting** | | **0.79** | **0.85** | **0.85** | **0.86** | **7** |
| | Neural Networks | | 0.79 | 0.78 | 0.76 | 0.74 | 7 |

| Rank | Feature | Category | Comment |
|---|---|---|---|
| 1 | Octane, 2-Methyl- | VOCs | Hydrocarbons |
| 2 | Benzaldehyde | VOCs | Aldehydes |
| 3 | Furfural | VOCs | Aldehydes |
| 4 | Hexanoic Acid | VOCs | Fatty acids |
| 5 | Geranyl Acetone | VOCs | Ketones |
| 6 | Sulfide, Allyl Methyl | VOCs | Sulphur-containing VOCs |
| 7 | 2-Nonanone | VOCs | Ketones |

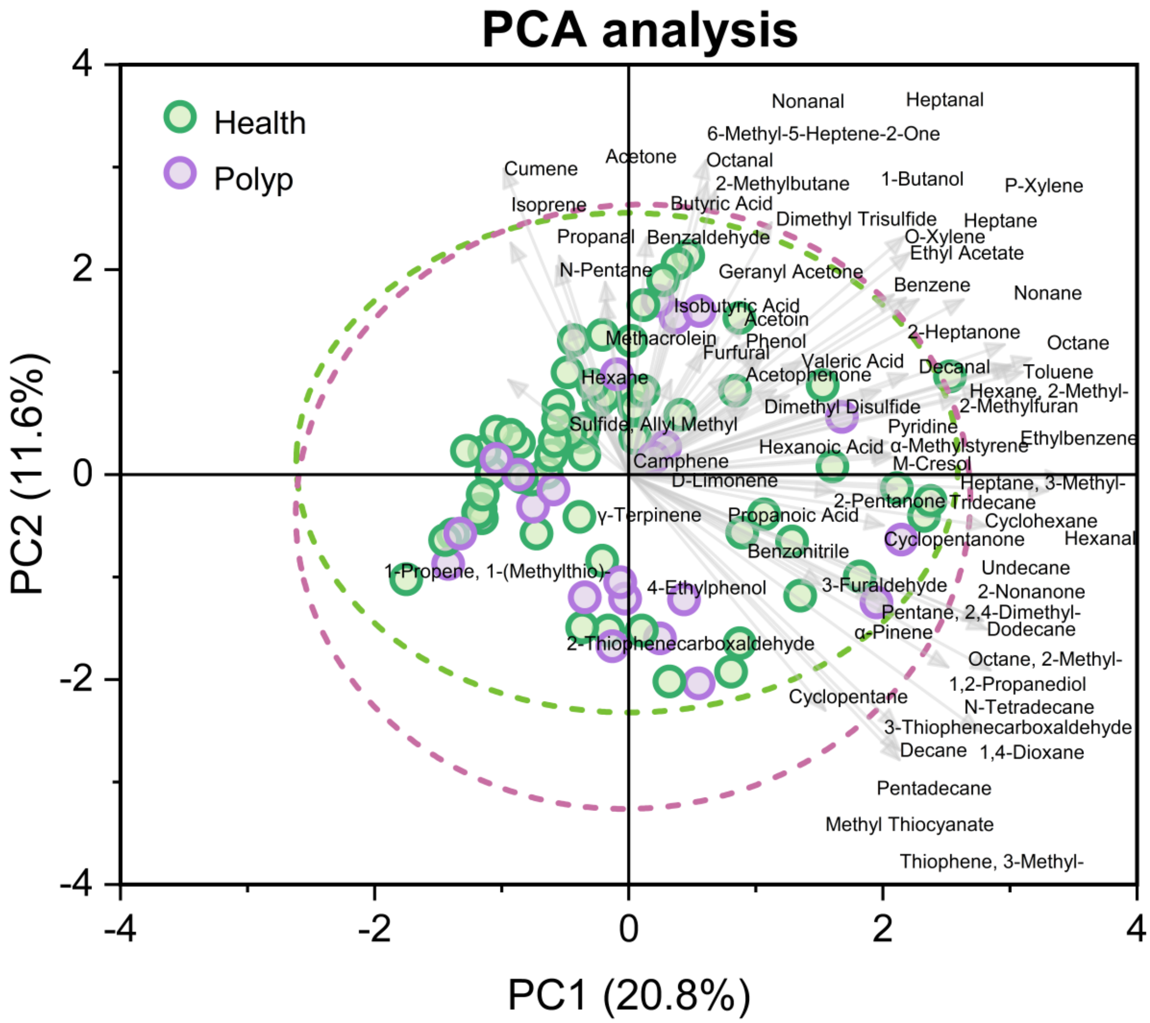


**Figure S1. PCA analysis of VOCs between polyp and completely healthy groups.**

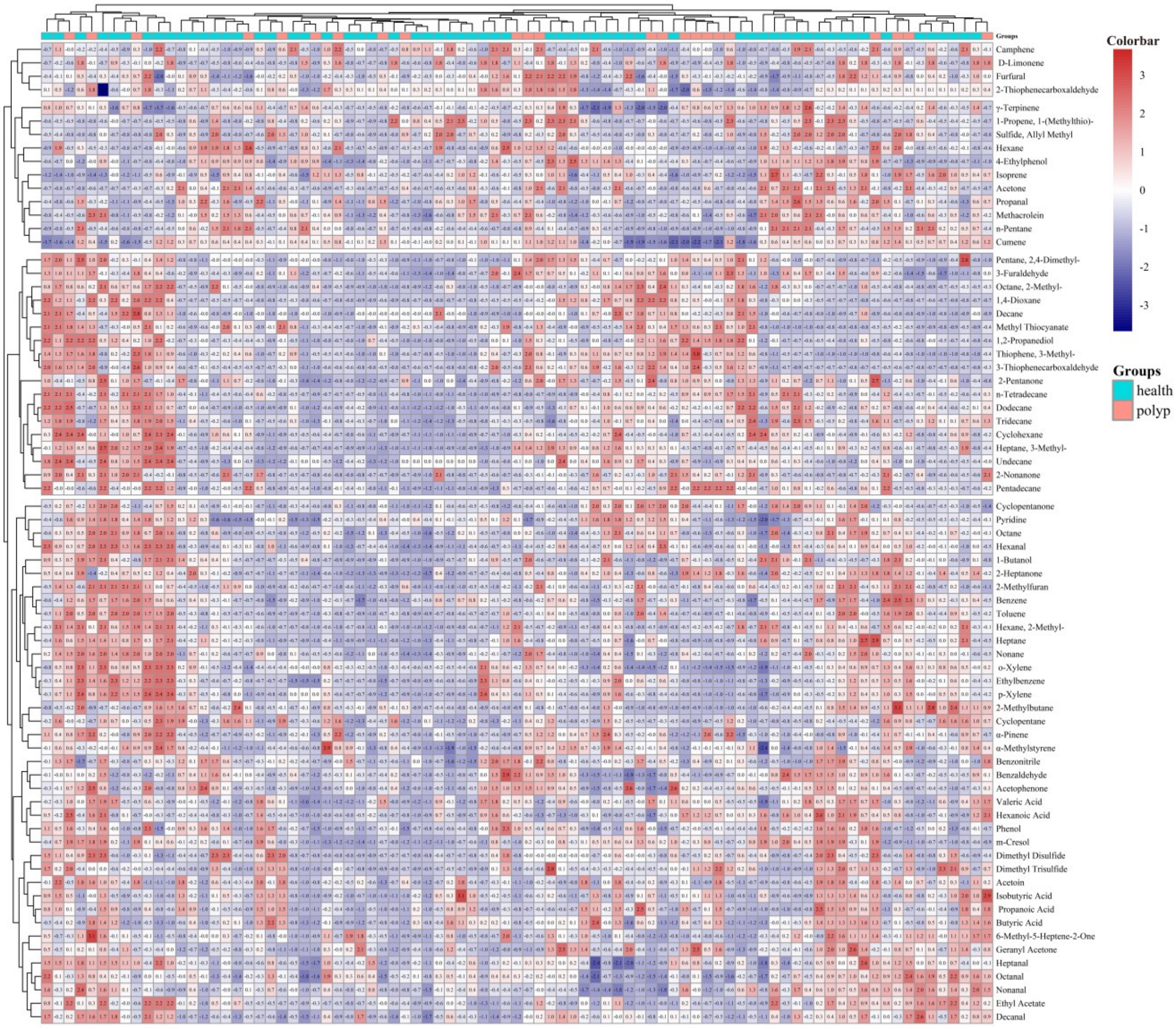


**Figure S2. Clustered heatmap of VOCs concentration between polyp and completely health groups.**

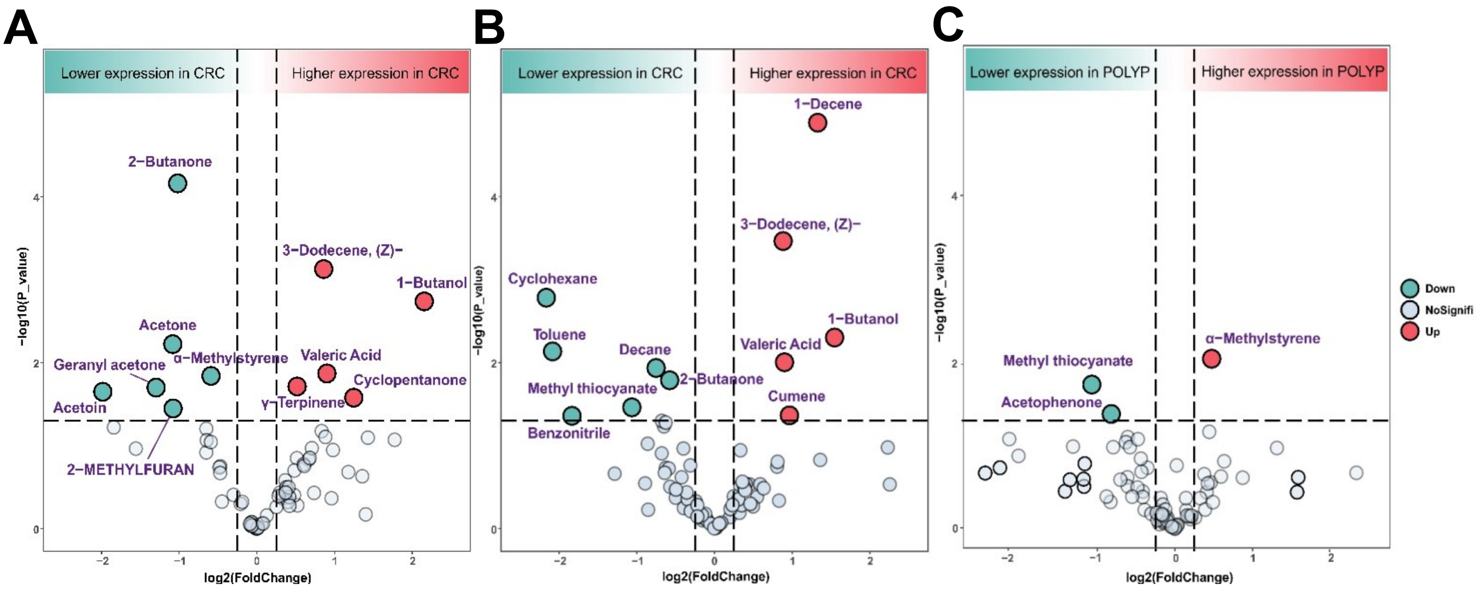


**Figure S3. Volcano diagrams of different groupings of substances in human exhalation. A.** CRC group versus healthy group. **B.** CRC group versus polyp group. **C**. healthy group versus polyp group.

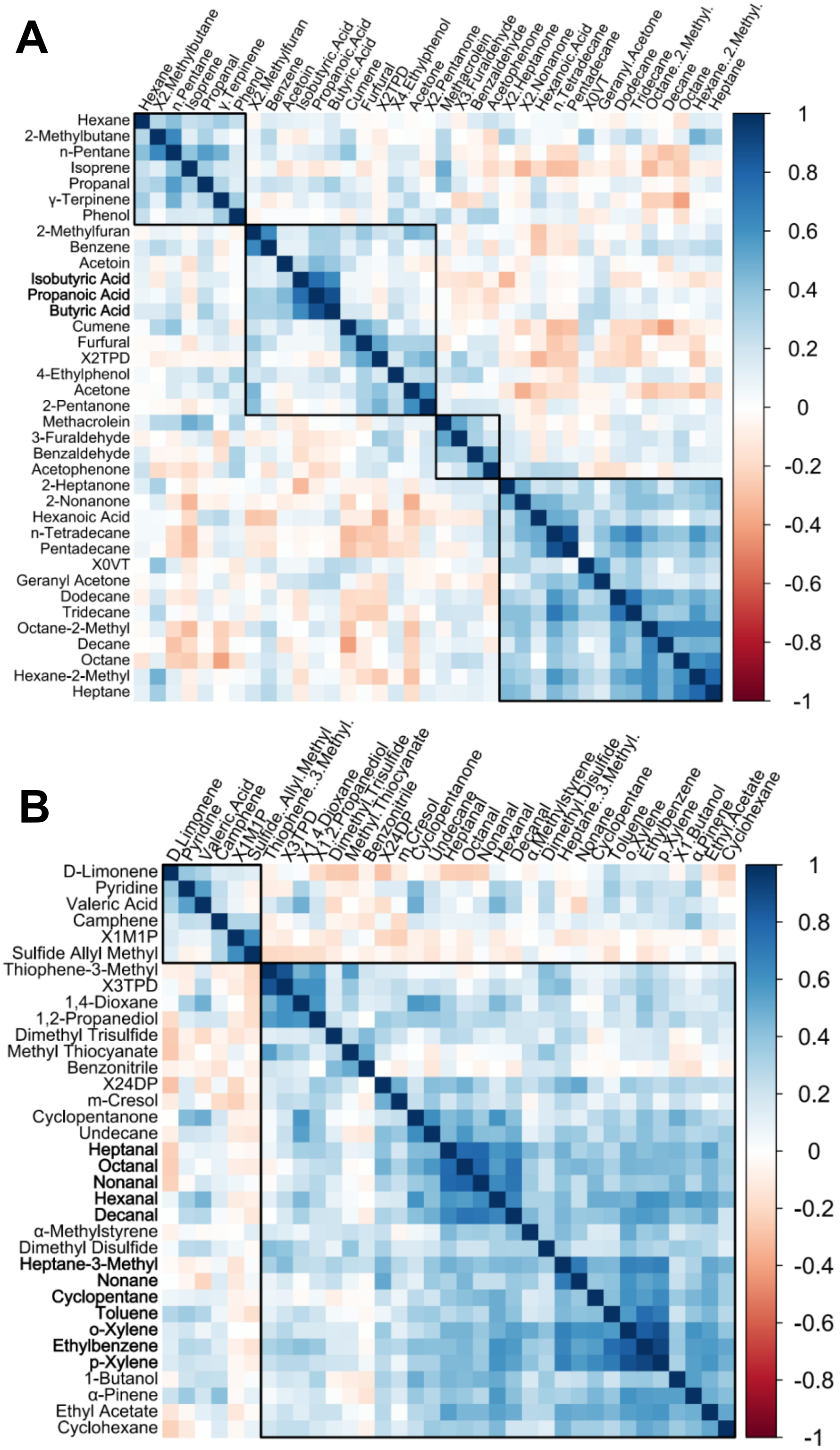


**Figure S4. Heatmaps of VOCs correlations in CRC. A.** A heatmap of the correlation of the first 37 substances and the formation of 4 large clustering modules. **B.** Correlation heatmap of the last 35 substances and formed 2 large clustering modules. Red represents negative correlation, blue represents positive correlation, and light white represents essentially no correlation.

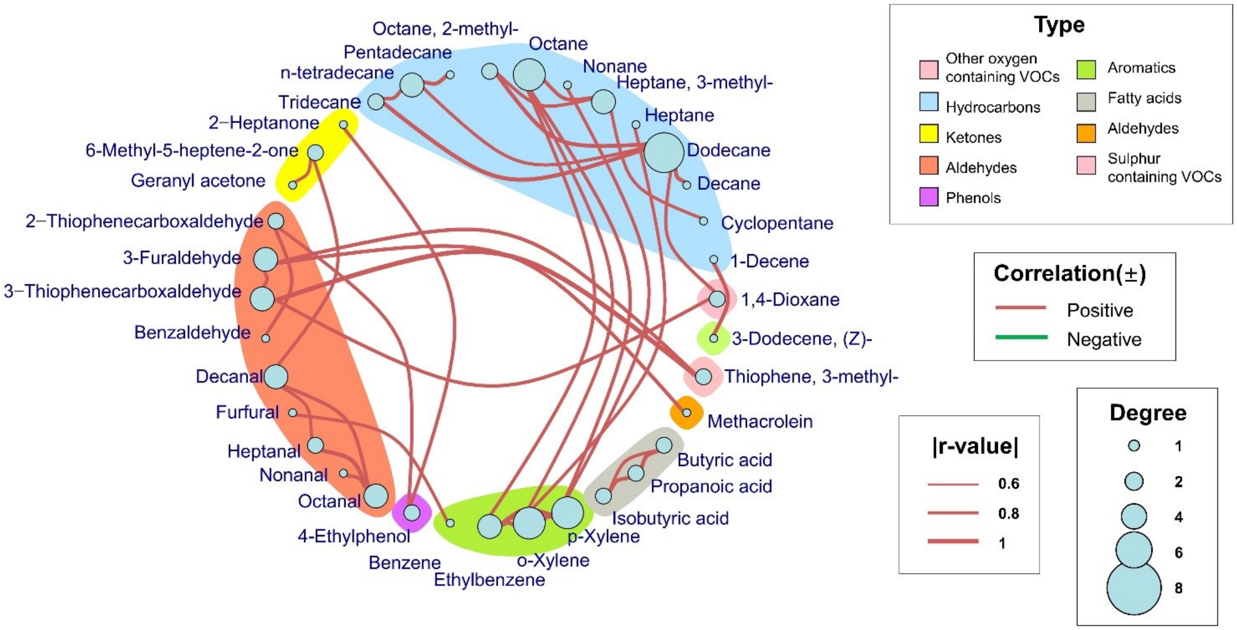


**Figure S5. Categorical relevance calculations by compounds groups.**

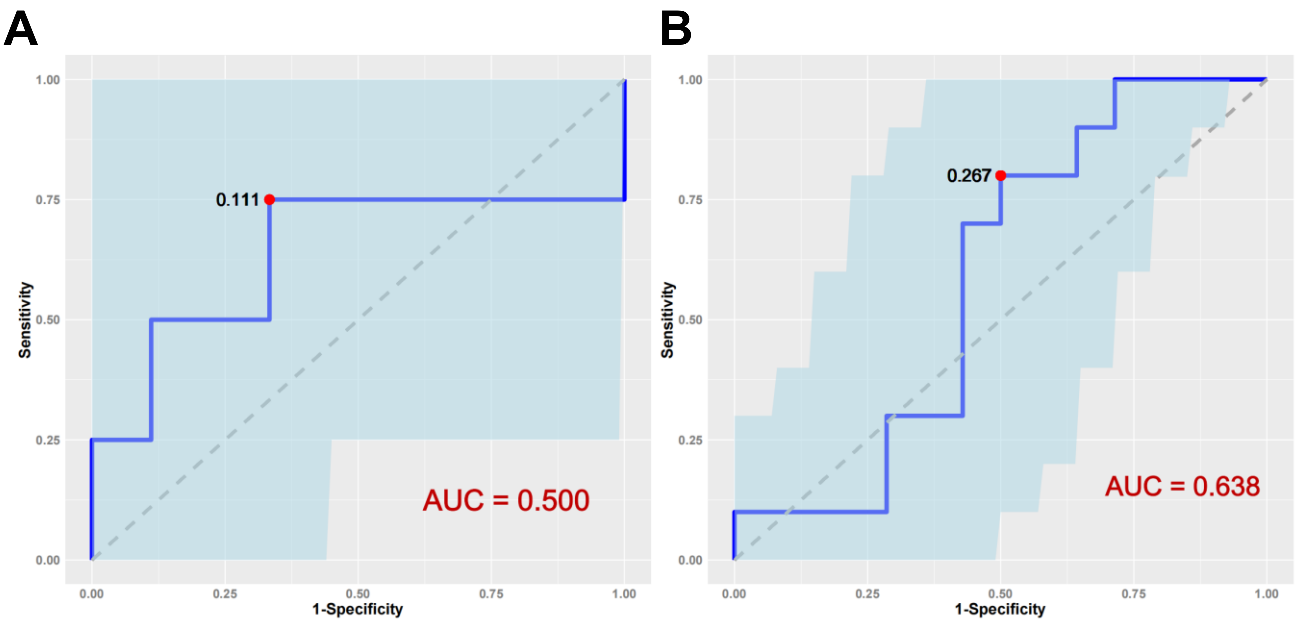


**Figure S6. The performance of Diagnostic Model in discerning LNM and DM from NM group. A.** distinguishing LNM from NM. **B.** distinguishing DM from NM.

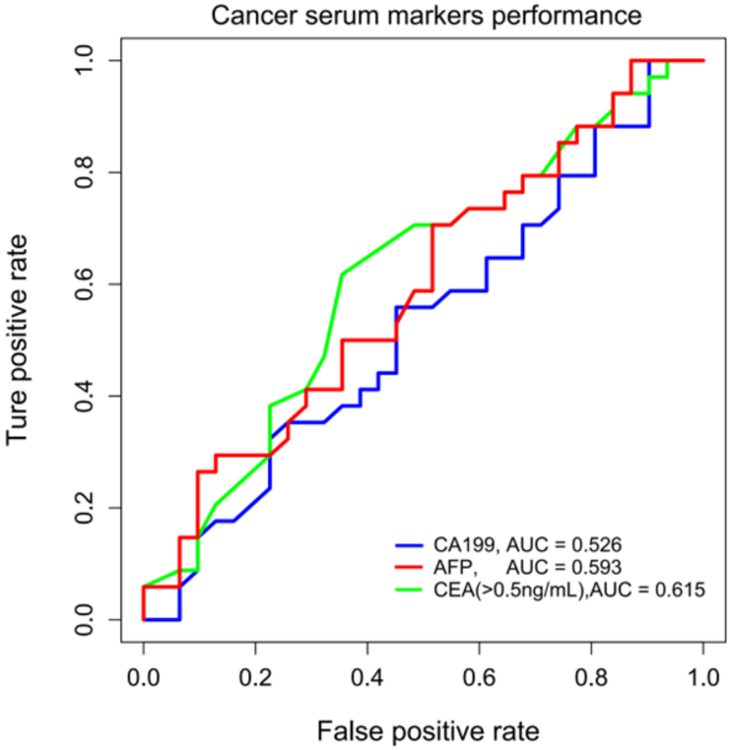


**Figure S7. The performance of traditional cancer serum markers.**

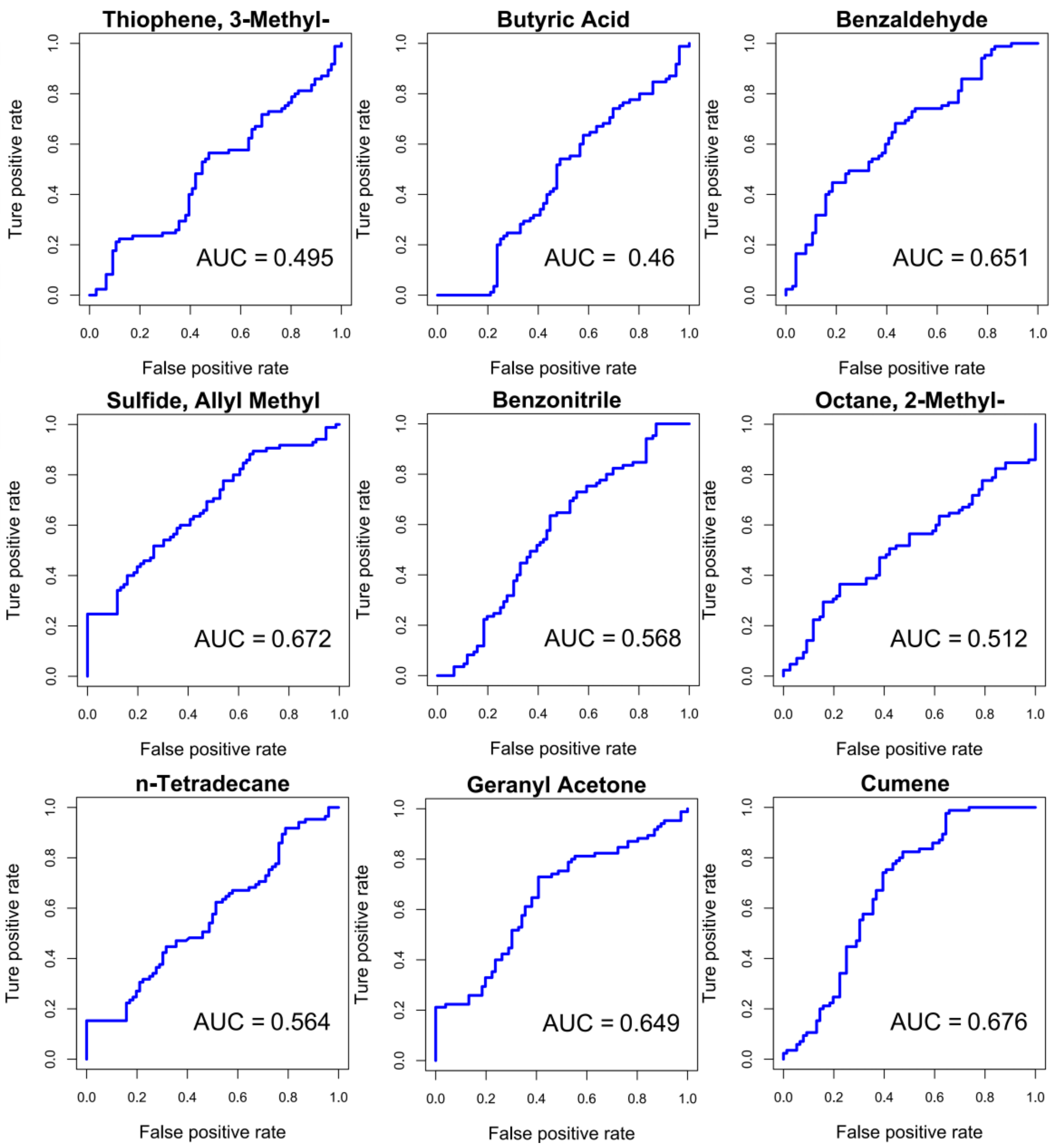
Thiophene, 3-Methyl-
AUC = 0.495
Butyric Acid
AUC = 0.46
Benzaldehyde
AUC = 0.651
Sulfide, Allyl Methyl
AUC = 0.672
Benzonitrile
AUC = 0.568
Octane, 2-Methyl-
AUC = 0.512
n-Tetradecane
AUC = 0.564
Geranyl Acetone
AUC = 0.649
Cumene
AUC = 0.676
Ture positive rate
False positive rate
0.0
0.2
0.4
0.6
0.8
1.0

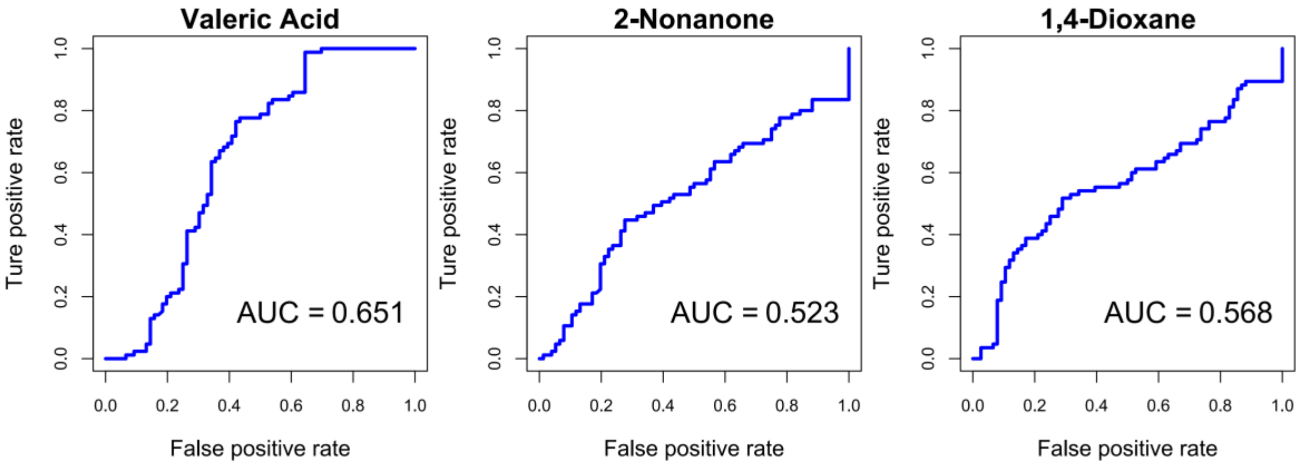


**Figure S8. The diagnosis efficiency of each marker among the fifteen markers in the diagnostic model.** ROC curves for each marker in the training cohort.

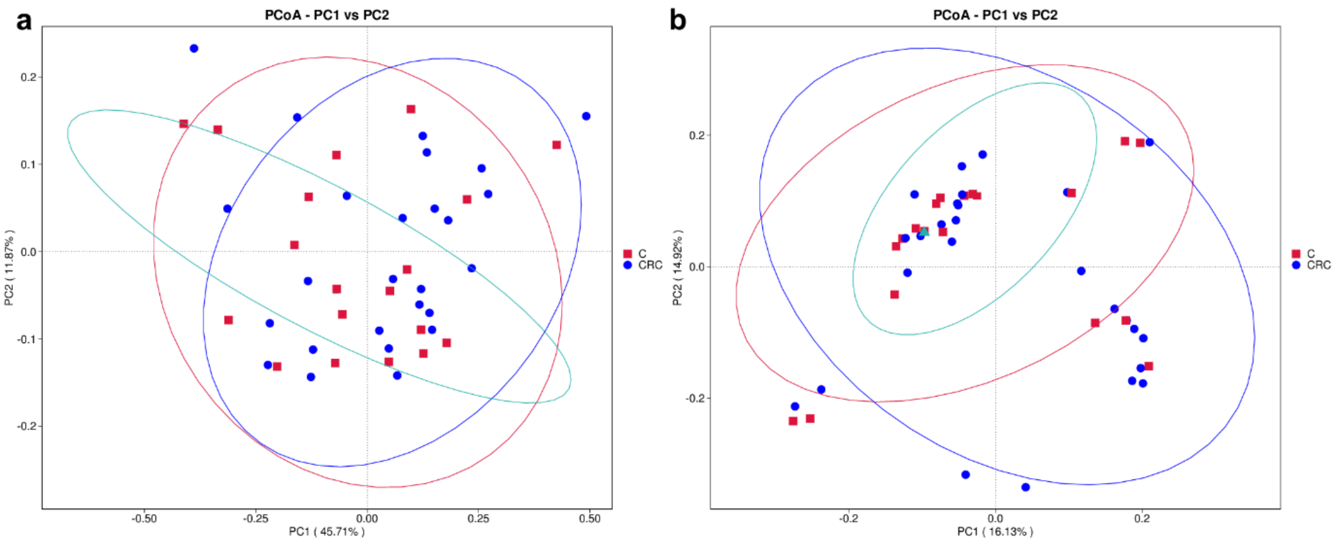


**Figure S9. Principal Co-ordinates Analysis (PCoA) Plots. A.** PCoA of a weighted UniFrac distance analysis. **B.** PCoA of an unweighted UniFrac distance analysis. Red for control, blue for CRC.

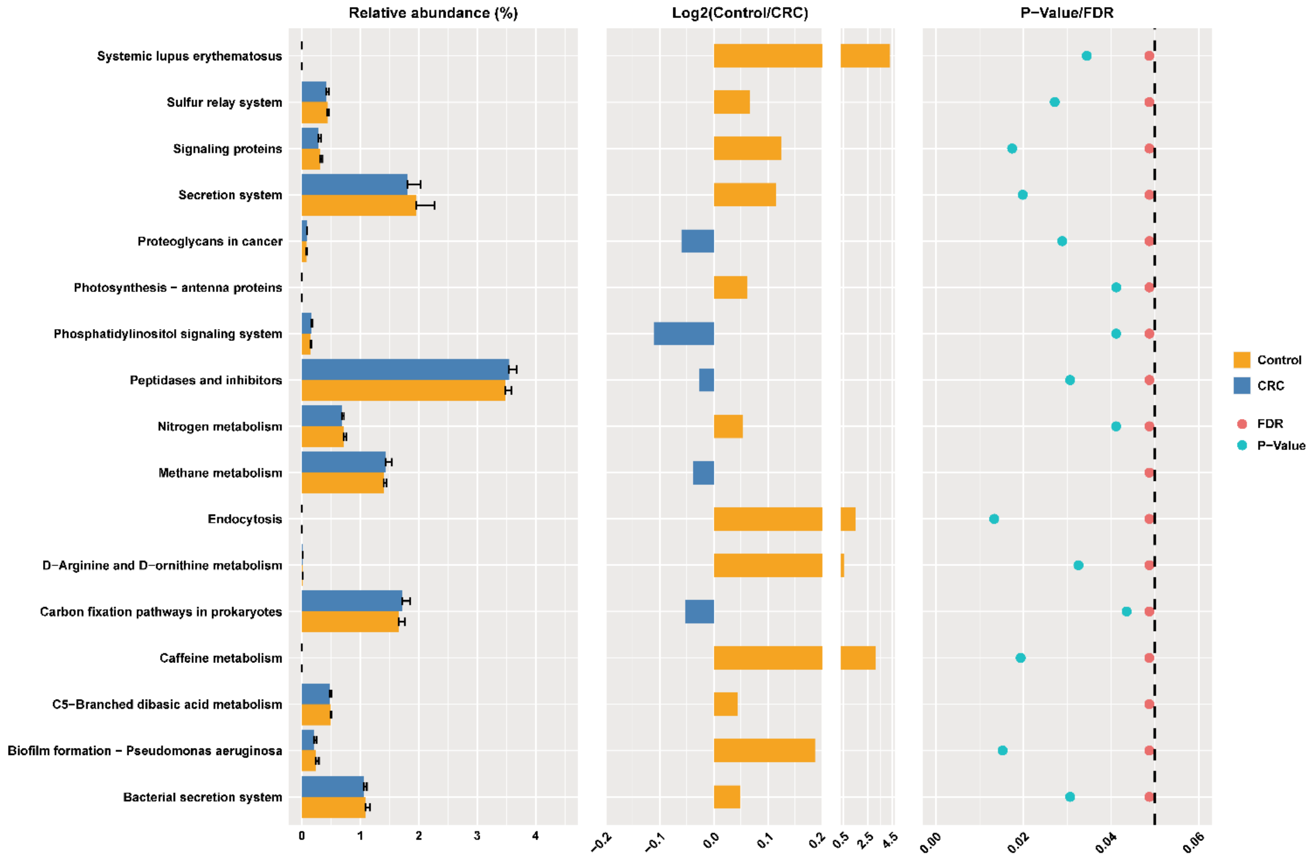


**Figure S10. Seventeen statistically tested significantly different KEGG pathways.** The left side of the figure shows the relative content gap between groups, the fold change value in the center, and the p-value and adjusted p-value on the right side. Orange for control, blue for CRC.

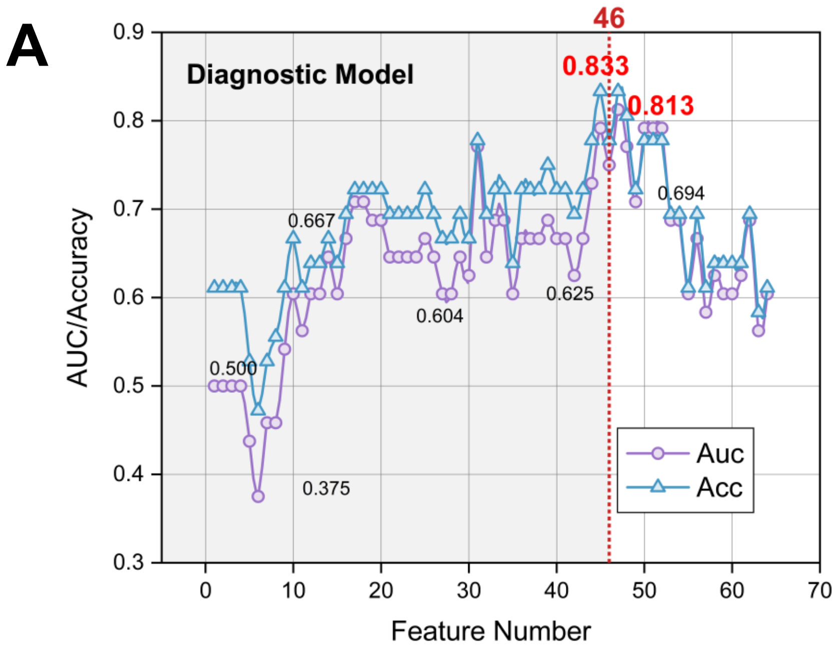


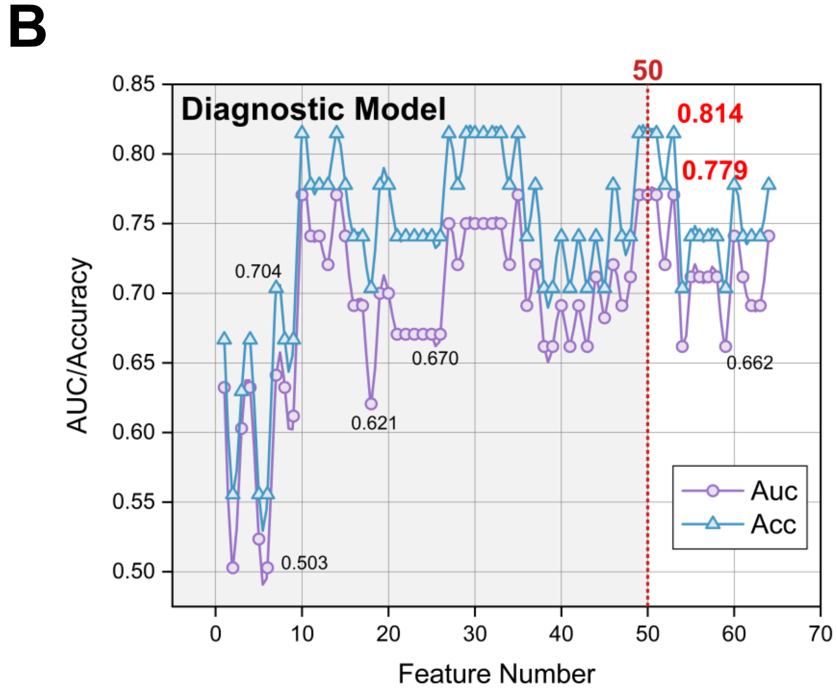


**Figure S11. The number of feature selection was determined by AUC and accuracy. A.** Baseline modeling with linear regression algorithm (64 features). **B.** Random Forest algorithm as a baseline model (64 features).

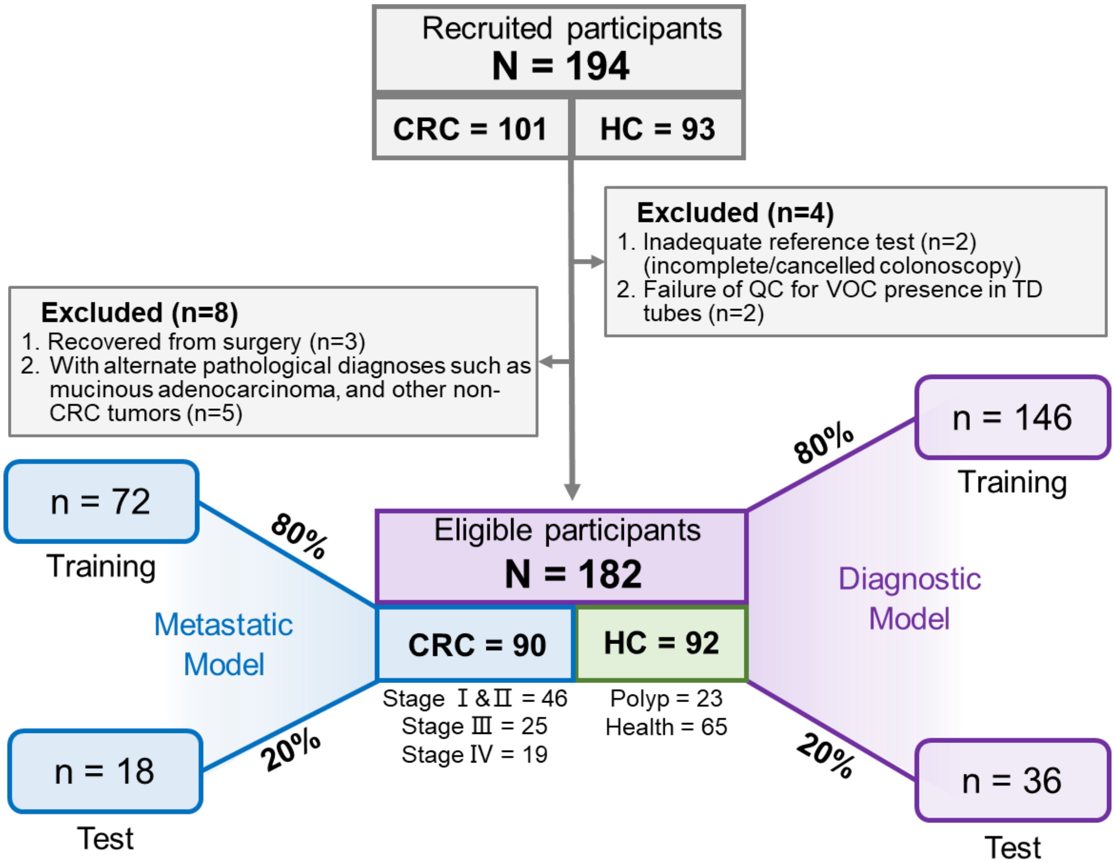


**Figure S12. Diagram of participant recruitment and allocation ratios.** The section in gray illustrates the process of excluding ineligible participants, and the section in color illustrates the process of assigning the 182 eligible participants in the model construction.
